# Tunicate bulb size variation in monocots explained by temperature and phenology

**DOI:** 10.1002/ece3.5996

**Published:** 2020-02-27

**Authors:** Cody Coyotee Howard, Nico Cellinese

**Affiliations:** ^1^ Florida Museum of Natural History University of Florida Gainesville Florida; ^2^ Department of Biology University of Florida Gainesville Florida; ^3^ Biodiversity Institute University of Florida Gainesville Florida; ^4^ Genetics Institute University of Florida Gainesville Florida

**Keywords:** geophytes, hysteranthy, monocots, synanthy, tunicate bulbs, underground storage organs

## Abstract

Plant bulbs are modified shoot systems comprised of short internodes with apical bud(s) surrounded by layers of leaf bases. Bulb diameters can vary greatly, with overall bulb size playing a role in flower formation and resource allocation. Despite the importance of bulb size to the overall fitness of an individual, evolutionary and ecological aspects of this trait have been almost completely neglected. Examining over 2,500 herbarium vouchers for 115 selected species, we analyzed monocot tunicate bulb size within a phylogenetic context in order to investigate its evolutionary significance. We recorded two bulb diameter optima and observed that as bulb size increases taxa inhabit warmer areas with less temperature seasonality. Furthermore, we found that hysteranthous taxa, a habit where leaves emerge separately from flowers, exhibit overall larger bulbs potentially due to reliance upon belowground stored resources to flower rather than on current environmental inputs. This work highlights the importance of including the belowground portion of plants into ecological and evolutionary studies in order to gain a more complete understanding of the evolution of plant forms and functions.

## Graphical abstract

Utilizing herbarium specimen data, we test hypotheses related to tunicate bulb size variation across the monocots. We found that as bulb size increases, taxa tend to inhabit warmer, less thermally variable habitats. We also found that hysteranthous taxa (i.e., those that flower without leaves present) have larger bulbs relative to synanthous taxa (i.e., flower and leaves appear simultaneously).

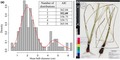

## 1 INTRODUCTION

Size plays a critical role in a number of physiological, developmental, ecological, and evolutionary processes across life's domains, with consequences at all levels of biological organization (Ackerly & Donoghue, 1998; Baker, Meade, Pagel, & Venditti, 2015; Hone & Benton, 2005; LaBarbera, 1986; Peters, 1983; Pimiento, Cantalapiedra, Shimada, Field, & Smaers, 2019; Rees, 1996; Testo & Watkins, 2012; Zotz, Hietz, & Schmidt, 2001). In animals, size differences correlate with prey selection (Boback, 2003; Deangelis & Coutant, 1982; Pimiento et al., 2019), mating and fighting tactics (Emberts, Miller, Li, Hwang, & St. Mary, 2017; Lailvaux, Herrel, Vanhooydonck, Meyers, & Irschick, 2004), metabolism (Gillooly, Brown, West, Savage, & Charnov, 2001; Reich, Tjoelker, Machado, & Oleksyn, 2006), ecological niches (Church, Donoughe, Medeiros, & Extavour, 2019; Pimiento et al., 2019), and fitness (Mammola, Milano, Vignal, Andrieu, & Isaia, 2019; Ollerton & Lack, 1998). In plants, cell size is dictated by genome size, and the increase in genome size can then act as a constraint on the rate at which physiological processes, such as mitosis and photosynthesis, can occur (Beaulieu, Leitch, Patel, Pendharkar, & Knight, 2008; Grime & Mowforth, 1982; Knight, Molinari, & Petrov, 2005). Within populations, larger plants produce flowers earlier and for longer durations, often have larger seeds, and produce a greater number of leaves, flowers, and fruits (Albert, Iriondo, Escudero, & Torres, 2008; Bustamante & Búrquez, 2008; Han, 2001; Marquis, 1988; McIntosh, 2002; Ollerton & Lack, 1998; Rees, 1969, 1996; Susko & Lovett‐Doust, 2000). However, studies on interspecific variation show larger (i.e., taller) plants flower later relative to smaller plants (Huang, Koubek, Weiser, & Herben, 2018), suggesting different life‐history tradeoffs at different ecological scales. Therefore, investigations into the effects of size at different scales (e.g., within and among populations, between closely and distantly related groups, or cellular vs. morphological) can provide insights into the strategies that both small and large organisms adopt, and the subsequent ecological and evolutionary consequences of such changes.

Geophytes, plants with buds located belowground on structures such as stem tubers, bulbs, corms, and rhizomes (e.g., potato, onion, crocus, and ginger), make for an interesting study system when investigating the macroevolutionary processes promoting and constraining plant size. Many taxa can accumulate sometimes large amounts of carbohydrates and/or water in belowground organs (Al‐Tardeh, Sawidis, Diannelidis, & Delivopoulos, 2008; Boeken, 1990; Ranwala & Miller, 2008; Ruiters, 1995; Veselý, Bureš, & Šmarda, 2013). Therefore, conceptually, the larger the organ, the greater resource storage capacity a taxon retains. This accumulation of resources can allow for greater independence from environmental constraints, such as precipitation, as well as greater buffering capacity against resource fluctuations (Dafni Cohen & Noy‐Mier, 1981; Dafni Shmida & Avishai, 1981; Procheş, Cowling, & Preez, 2005). Underground storage organ (USO) size influences several life‐history processes as well, such as leaf emergence, flowering, and seed set (Dafni, Cohen, et al., 1981; Dafni, Shmida, et al., 1981; Han, 2001; Hertogh, 1996; Rees, 1966, 1969, 1972). Belowground reserves can be drawn upon to divide apical cells during the dormant season in order to rapidly fill these cells at the onset of the growing season (Grime & Mowforth, 1982), which can allow for relatively earlier emergence, and maximum capture of available resources (e.g., light, water) that are typically in short supply, given the highly seasonal climates that many geophytic taxa inhabit (Cuéllar‐Martínez & Sosa, 2016; Howard, Folk, Beaulieu, & Cellinese, 2019; Rees, 1989). USO size can also be used as a proxy for flowering (i.e., once a bulb is a certain diameter it should flower) (De Mastro & Ruta, 1993; Han, 2001; Hanzawa & Kalisz, 1993), although adequate USO size must be obtained before flowering can occur (Dafni, Cohen, et al., 1981; Dafni, Shmida, et al., 1981; Hanzawa & Kalisz, 1993). Therefore, there is no doubt USO size plays a critical role in geophyte evolution and ecology.

To date, only a few studies have focused on the ecological significance of USO size (Dafni, Cohen, et al., 1981; Dafni, Shmida, et al., 1981; Procheş et al., 2005). Dafni, Cohen, et al. (1981) proposed that two phenological patterns are tied to USO size: synanthy and hysteranthy. Synanthy is the process involving leaves and flowers emerging simultaneously, whereas in hysteranthy, flower and leaf emergence are temporally separated (Dafni, Cohen, et al., 1981; Dafni, Shmida, et al., 1981). These distinct strategies determine whether or not leaves can be relied upon to fuel flowering, fruiting, and seed set. Consequently, synanthous taxa may not be able to emerge until conditions are conducive for growth (e.g., no freezing temperatures, wet season) since leaves and/or roots may be more heavily relied upon to supplement USO reserves during growth. Since leaves are typically not present during flowering in hysteranthous taxa, this may require more sufficient belowground reserves to fuel flowering and fruiting (Dafni, Cohen, et al., 1981). Therefore, hysteranthous taxa likely need larger USOs compared to synanthous taxa, and require longer, more reliable growing seasons to replenish and maintain larger USOs (Dafni, Cohen, et al., 1981; Dafni, Shmida, et al., 1981). Currently, there is little support for these hypotheses, and these processes have not been the object of active research.

Among monocotyledonous geophytes, bulbous plants generally inhabit some of the coldest and/or driest climates (Howard et al., 2019; Patterson & Givnish, 2002). Therefore, the evolutionary and ecological consequences of bulb size may play a more critical role in their survival relative to other geophytic taxa. The plant bulb is a modified shoot system consisting of a compressed stem with short internodes surrounded by concentric layers of leaf bases, which are where nutrient and water storage occurs (Al‐Tardeh et al., 2008; De Hertogh & Nard, 1993; Rees, 1972; Ruiters, 1995). The vast majority of bulbous monocots are tunicate bulbs (Rees, 1972), which retain the outer, dried layers of leaf bases (i.e., the tunica; e.g., the dried outer layers of an onion) that are thought to improve water retention and/or provide protection from external factors, such as soil shrinkage due to drought, while dormant belowground (Al‐Tardeh et al., 2008). Nontunicate or imbricate bulbs are predominantly found in the *Lilium* + *Nomocharis *+ Fritillaria clade (~300 taxa; Liliales) and lack a tunica (Patterson & Givnish, 2002; Rees, 1972), leaving bulbs more exposed to external influences. Testing fundamental hypotheses related to the evolution of tunicate bulb size is critical but completely lacking. Understanding the historical processes that have constrained and/or promoted size diversity in bulbs will lend further insights into the evolutionary implications of size shifts across plant organs.

In this study, we focus our investigation specifically on tunicate bulb size within the monocots. We exclude taxa with imbricate bulbs (i.e., *Lilium* + *Nomocharis *+ Fritillaria clade) because the absence of a tunica implies lack of constraints on the bulb's outward growth and significant ecological ramifications (e.g., they inhabit narrower niche spaces; Figure S1). Additionally, all taxa in this clade exhibit a consistent synanthous phenology and during the preservation process, they are significantly crushed and inconsistently flattened on herbarium sheets. Therefore, standardizing an appropriate approach to measure their diameter is a very difficult proposition.

Using herbarium specimens, we measure tunicate bulb size in order to (A) examine how some ecological factors may have influenced size variation and (B) test whether different phenological patterns are correlated with differences in size. We hypothesize the following:

1. Taxa with smaller bulbs can withstand less climatic variation compared to those with larger bulbs, since smaller reserves are available for sustainability and/or survival should unfavorable conditions persist for consecutive growing seasons.
2. Synanthous taxa possess relatively smaller bulbs compared to hysteranthous taxa, since reliance upon stored resources for flowering is not as strong.
3. Hysteranthy will require larger USOs to fuel flowering. These larger USOs have been coopted to allow taxa to inhabit perhaps more variable climates, since stored resources can be utilized to fuel growth during less‐than‐optimal seasons (i.e., they can reliably produce leaves or flowers during such times).

## 2 METHODS

### 2.1 Herbarium voucher selection, measurement, and taxon coding

**Figure 1:**
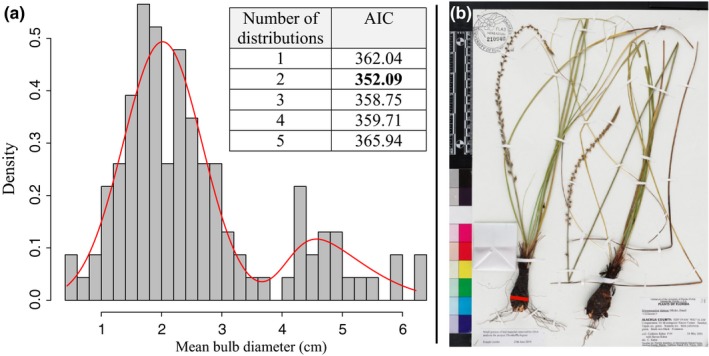
Digitized specimens have become a powerful resource for uncovering interesting ecological and evolutionary patterns. We found that tunicate bulb size exhibits a bimodal distribution (a). Histogram and density plot of mean bulb diameter (cm) (a) measured from 115 herbarium vouchers of tunicate bulbous taxa (b). Inset table in “a” shows that two modes are favored by AIC using finite mixture models. Red line in “b” demonstrates where measurements were taken on each acceptable, farthest left individual. Image University of Florida Herbarium specimen, FLAS 210940, Florida Museum of Natural History, by Kathy M. Davis on Friday, July 2, 2010

Recent calls to mass digitization of museum repositories have generated a deluge of available specimen data (Beaman & Cellinese, 2012). Subsequently, the use of digitized herbarium specimens has led to recent advances in understanding phenological shifts through time, range sizes, species richness, and morphological diversity across plant lineages (Soltis, 2017; Soltis, Nelson, & James, 2018). Utilizing digitized herbarium specimens, as data permitted, we selected 115 tunicate monocotyledonous bulbous species that represented both their phylogenetic (based on Howard et al. (2019)) and known morphological diversity (i.e., bulb diameter). Herbarium specimen images for each taxon were downloaded from the Global Biodiversity Information Facility (GBIF.org; see Appendix S1 for list of taxa and records used). Images were visually vetted for (a) the presence of a whole bulb and (b) a scale bar for size. An exception to the scale bar requirement was given to specimens from the Muséum National d'Histoire Naturelle in Paris (P) since barcode labels measuring 5 × 2 cm were found on each voucher (Marc Jeanson, pers. comm.) and used as reference. Specimens with visibly crushed bulbs, large portions of the bulb missing, and/or bulbs that were difficult to orient (i.e., could not distinguish the apical portion from the bulb base) were discarded. Additionally, vouchers with dubious species identification were also discarded. Of all the suitable specimens, 30 were randomly selected for each taxon and measurements of the bulbs were taken at the widest diameter (e.g., red line in Figure 1b). Taxa represented by less than 30 suitable vouchers were all measured. Bulbs are commonly sectioned in half prior to vouchering in order to aid the drying and mounting process, therefore, in order to avoid unintentionally measuring the same individual bulb twice (two equal halves), we only measured a single bulb from each herbarium specimen with multiple bulbs mounted on the same sheet. In order to improve reproducibility, the selection and measurement of bulbs were consistently performed on the farthest left acceptable bulb found on the specimen. Measurements were taken using ImageJ v. 1.52c (Abràmoff, Magalhães, & Ram, 2004). The mean value for each taxon was calculated and used in subsequent statistical and phylogenetic comparative analyses using R v. 3.5.3 (R Core Team, 2016) (see below). Lastly, each taxon was coded as either synanthous or hysteranthous based on available descriptive resources (Al‐Tardeh et al., 2008; de Andrade et al., 2012; Boeken & Guttermann, 1989; Dafni, Cohen, et al., 1981; Dafni, Shmida, et al., 1981; Daniels, Mabusela, Marnewick, & Valentine, 2013; Duncan, 2016; Hoffmann, Liberona, & Hoffmann, 1998; Snijman & Linder, 1996; Speta, 1998; Stedje, 1987) and/or herbarium vouchers. In some taxa, hysteranthy is facultative (Dafni, Cohen, et al., 1981; Dafni, Shmida, et al., 1981), resulting in herbarium vouchers with both leaves and flowers. However, in these cases, taxa were coded as hysteranthous since they are capable of this phenology. If no mention of hysteranthy was associated with a taxon, it was coded as synanthous upon exhaustive literature search.

### 2.2 Statistical (nonphylogenetic) analyses

To obtain optimum value(s) for bulb diameter, we used a finite mixture model available from the R package mixsmsn v. 1.1‐5 (Prates, Cabral, & Lachos, 2013) using the mean value for each taxon as input. These sets of analyses allow for the fitting of probability densities on skewed distributions, which our data exhibited (Figure 1a). Since we had no prior expectations on bulb sizes, we fitted 1–5 skew normal distributions to the data, using a maximum of 1,000 iterations. In order to determine the optimal number of modes and their approximate peak values, models were compared using Akaike information criterion (AIC). The lowest AIC was used to determine the best model fit.

### 2.3 Phylogenetic reconstruction and time calibration

In order to incorporate a phylogenetic correction in subsequent analyses (see next section), we needed an ultrametric tree. We used the web‐based platform OneTwoTree (Drori et al., 2018; website http://onetwotree.tau.ac.il/), which retrieves sequences from GenBank (Benson, Lipman, & Ostell, 1993) for a predetermined list of taxa, places these sequences into orthologous groups, chooses the most informative markers, and then performs phylogenetic reconstruction on a partitioned sequence supermatrix using maximum likelihood or Bayesian inference (Drori et al., 2018). We selected the 115 taxa measured for bulb diameter as input. Phylogenetic reconstruction using maximum likelihood (RAxML; Stamatakis, 2014) was run with 1,000 bootstrap replicates. Defaults were used for all other parameters. In addition to the bulbous taxa, 51 additional taxa were included to accommodate fossil calibration points (Figure S2). Time calibration was performed using penalized likelihood as implemented in treePL (Smith & O'Meara, 2012). The following calibration points were as follows: (a) a fossil dated between 48.88 and 49.96 MYA at the crown of Amaryllidaceae (Pigg, Bryan, & DeVore, 2018), (b) a fossil dated between 33.8 and 34 MYA placed at the crown of Alismataceae (Iles, Smith, Gandolfo, & Graham, 2015), (c) a fossil dated between 72.1 and 83.6 MYA placed at the crown of Zingiberales (Iles et al., 2015), (d) a fossil dated at 23.2 MYA at the crown of Asteliaceae, (e) a fossil dated between 14.5 and 16.2 MYA at the crown of Agavoideae, (f) a secondary calibration of 133–136 MYA at the split between *Acorus calamus* and the remaining monocots (Givnish et al., 2018), and (g) a secondary calibration of 136–139.35 MYA at the split between *Amborella trichopoda* and the remaining angiosperms (Magallón, Gómez‐Acevedo, Sánchez‐Reyes, & Hernández‐Hernández, 2015). One priming step followed by 10 cross‐validations was performed in order to obtain the appropriate smoothing parameter of 0.1. For the final dating analysis, 500,000 penalized likelihood iterations and 100,000 cross‐validation optimization iterations were used.

### 2.4 Climate data acquisition and correlations with bulb size

We determined the best model of evolution for bulb size by fitting four different models (i.e., Brownian motion, Orstein–Uhlenbeck [OU], white noise, and early burst) across the phylogeny and selecting the one with the lowest AIC score. This was accomplished using the fitContinuous function in phytools v 0.6‐44 (Revell, 2012). In order to understand how bulb size has been shaped by climate, we obtained climatic data using geospatial coordinates downloaded from GBIF (http://www.gbif.org) for each taxon. Occurrence records were downloaded using the R package rgbif v. 1.3.0 (Chamberlain, Ram, Barve, Mcglinn, & Chamberlain, 2016), and duplicate locations for each taxon were removed. Climate data were obtained by using a custom Python script developed by R.A. Folk (website https://github.com/ryanafolk/Saxifragales_spatial_scripts/tree/master/Extract_point_values). This script avoids oversampling of each taxon within the same grid cell. Mean values for each climatic variable for each taxon were calculated. Highly correlated climatic variables were removed from subsequent analyses using the R package caret v. 6.0‐81 (Kuhn et al., 2015) with a correlation cutoff of 80%. Using the R packages nlme v. 3.1‐137 (Pinheiro, Bates, DebRoy, Sarkar, & Team, 2013) and ape v. 5.3 (Paradis, Claude, & Strimmer, 2004), we investigated correlations between bulb diameter and climate. The uncorrelated climatic variables were analyzed within a phylogenetic framework using phylogenetic generalized least squares (PGLS) assuming the best model of evolution as determined from above (i.e., OU, AIC = 280.52; Table S1). A square root transformation was applied to bulb diameter in order to better meet the assumptions of the model (e.g., normality). In addition to the climatic variables, we also accounted for phenology (i.e., synanthous vs. hysteranthous). This resulted in a model that included bulb size and ten explanatory variables (i.e., phenology, BIO2 [mean diurnal range], BIO4 [temperature seasonality], BIO5 [max temperature of the warmest month], BIO8 [mean temperature of the wettest quarter], BIO9 [mean temperature of the driest quarter], BIO15 [precipitation seasonality], BIO17 [precipitation of the driest quarter], BIO18 [precipitation of the warmest quarter], and BIO19 [precipitation of the coldest quarter]). Using the R package MASS v. 7.3‐51.1(Venables & Ripley, 2002), model selection using both forward and backward step AIC was implemented to determine the best model among the different combinations of bulb size, phenology, and climate.

## 3 RESULTS

### 3.1 Bulb size modality

The minimum number of individual bulb measurements for a taxon was two (i.e., *Gethyllis spiralis*, likely due to low representation in collections, given it is a South African endemic restricted to small, sandy areas of the Cape Province; Figure S3). We noticed that larger bulbs were less represented on specimens (i.e., only aboveground parts were present), which highlights the need for increased preservation of larger USOs in herbarium collections. Surprisingly, the mean values for each taxon show a clear bimodal distribution for bulb size (Figure 1a), which significantly deviates from a normal distribution (Lilliefors normality test: *p* = 1.053e‐06). Finite mixture models favored (via AIC) two skew normal distributions with peak mode values of approximately 2.38 and 4.18 cm (Figure 1a). The use of median returned qualitatively similar results (i.e., bimodality, AIC = 344.29, mode peak values of 1.51 and 4.13 cm; data not shown). Individual measurements for each taxon with GBIF identification numbers can be found in Digital Dryad (https://doi.org/10.5061/dryad.sf7m0cg25).

### 3.2 Bulb size evolution and ecology

Of the 166 taxa used as input into OneTwoTree, 145 had sufficient GenBank data and were included in the phylogenetic reconstruction. The concatenated supermatrix alignment was comprised of 16 loci with 32,925 base pairs. Phylogenetic relationships agree with our current understanding of the monocot phylogeny (Givnish et al., 2018; Howard et al., 2019); however, we recover age estimates across the tree that vary in the degree of congruence with past studies (Figure S2), likely due to our widespread taxon sampling and/or age estimation methodology. Fortunately, PGLS analyses are robust to phylogenetic uncertainty (e.g., branch lengths; Díaz‐Uriarte & Garland, 1998; Stone, 2011), and thus, we included the phylogeny simply as a correction for the remaining post‐tree analyses. The resulting phylogeny from OneTwoTree and treePL can be found in Digital Dryad (https://doi.org/10.5061/dryad.sf7m0cg25).

**Figure 2:**
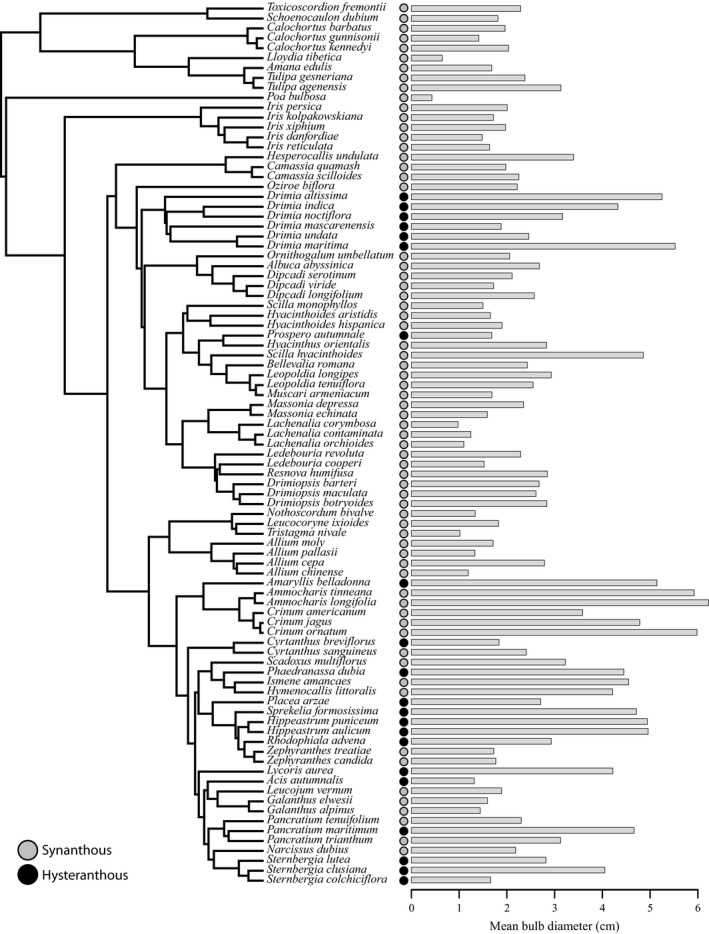
Phylogenetic relationships for 89 of the 115 tunicate bulbous taxa investigated with phenology coding and mean bulb diameter (cm) displayed at the tips

**Figure 3:**
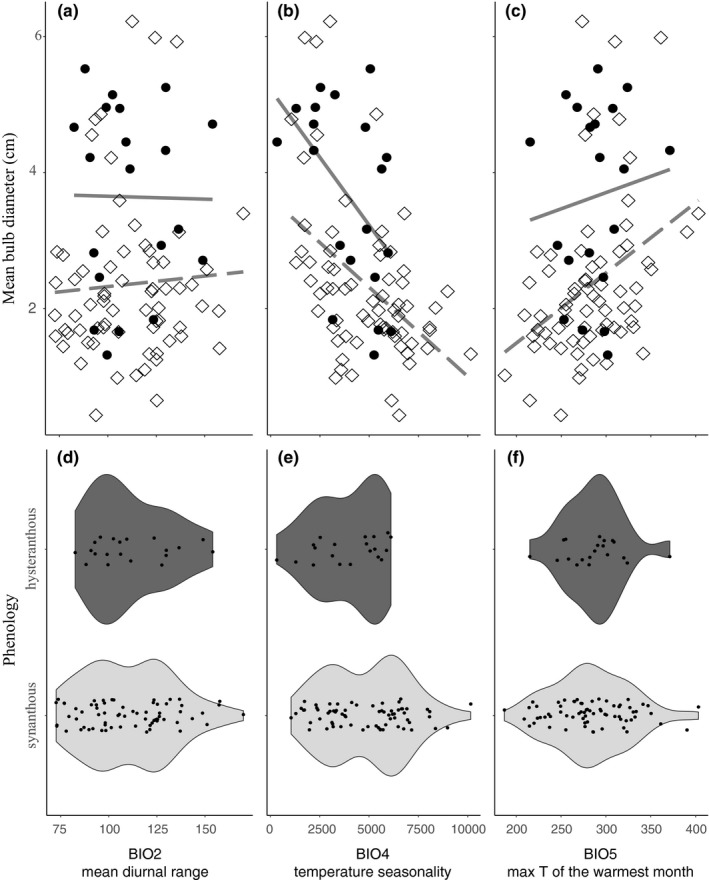
Of the 88 taxa with sufficient climate data, we see that hysteranthous taxa have larger bulbs and inhabit an overall reduced range of climate space relative to synanthous taxa. Graphical representation of the three best climate variables returned from model selection via step AIC. (a–c) Point coloration and shape as well as line regression type correspond to phenology (hysteranthous [black circles, solid line]; synanthous [white diamonds, dotted line]). (d–f) Violin plots showing the range of climate space occupied by hysteranthous (dark gray) and synanthous (light gray) taxa. Untransformed mean bulb diameter data displayed for ease of interpretation. BIO2 (mean diurnal range, a and d) and BIO5 (max temperature of the warmest month, c and f) expressed in degrees Celsius, BIO4 (temperature seasonality, b and e) expressed as a percentage

**Figure 4:**
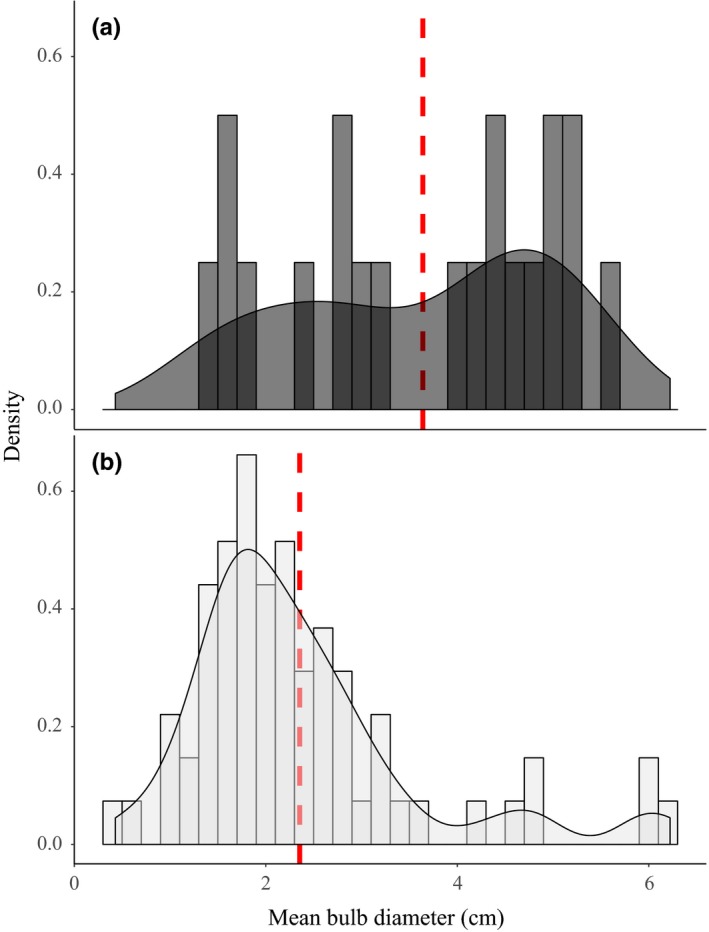
Overall, hysteranthous taxa have larger bulbs relative to synanthous taxa. Density plots of mean bulb diameter for the 115 taxa coded as either hysteranthous (a) or synanthous (b). The red dotted lines indicate the mean value for each phenology

Of the 115 bulbous taxa measured, 88 taxa had sufficient climate data for testing the relationship between bulb size, phenology, and climate (Figure 2). Of these, we found that 21 displayed a hysteranthous phenology (black circles, Figure 2). Using step AIC, the best model included phenology (synanthy vs. hysteranthy), mean diurnal range (BIO2), temperature seasonality (BIO4), and maximum temperature of the warmest month (BIO5) (AIC = 48.63; Figure 3; Table S2). These four variables were all significant, assuming an alpha cutoff of 0.05 (phenology: *p* = 3e10‐4; BIO2: *p* = .02, BIO4: *p* = 0, BIO5: *p* = 4e10‐4; Table S2). Our results suggest that taxa with larger bulbs generally inhabit warmer, more thermally stable climates (Figure 3a–c). Additionally, our results show that hysteranthous taxa occupy an overall reduced range of climate space relative to synanthous taxa (Figure 3d–f). Moreover, they possess, overall, larger bulbs (Figures 3a–c and 4a) and are found in warmer, less thermally variable climates relative to synanthous taxa (Figure 3a–c). Synanthous taxa inhabit a wider range of thermal niches and have relatively smaller bulbs (Figures 3 and 4b). Similarly to the overall bulb size trends, taxa with larger bulbs in both groups (i.e., hysteranthous vs. synanthous) appear to be more constrained to warmer, thermally stable climates (Figure 3a–c). Results when using median values returned qualitatively similar results (Figures [Link], [Link], [Link]). Climate data, mean measurements, and phenology scoring can be found in Digital Dryad (https://doi.org/10.5061/dryad.sf7m0cg25).

## 4 DISCUSSION

Studies investigating USO size variation have been limited (Dafni, Cohen, et al., 1981; Procheş et al., 2005). Here, we quantified variation in tunicate bulb size across a broad sampling of monocotyledonous taxa, and interestingly, we found that bulb size exhibits a bimodal distribution. Although it may intuitively seem advantageous to possess larger USOs, our results suggest that ecological constraints may affect variation in tunicate bulb size and that some taxa may have adopted alternative phenological strategies to capitalize on this size variation. Our data do not support the hypothesis that plants with smaller bulbs inhabit areas with less climatic variation (hypothesis 1). In fact, we show the inverse: plants with larger bulbs appear to inhabit more stable climates. Our results provide support for the hypothesis that plants with different phenological patterns exhibit variation in bulb size. That is, hysteranthous taxa have overall larger bulbs relative to synanthous taxa (hypothesis 2 and 3). This study provides a comparative baseline for future investigations on the ecology and evolution of bulb size, including size variation at both broad and fine scales.

Herbaceous plants in general tend to inhabit colder and/or drier habitats compared to their woody relatives (Zanne et al., 2014, 2018). Within herbaceous plants, those with buds buffered from the climate (e.g., by soil or leaf litter) typically inhabit even more extreme climates (Howard et al., 2019; Lubbe & Henry, 2019a,b; Sosa, Cameron, Angulo, & Hernández‐Hernández, 2016). For bulbous geophytes, our results suggest that taxa with larger bulbs appear to be best suited for warmer, more thermally stable climates. We observe this pattern since larger bulbs may require more inputs in order to (a) replace depleted nutrients used during dormancy, (b) allow enough time for growth and flowering, and (c) prepare for an upcoming dormant season. Although larger USOs may allow taxa to inhabit landscapes with more unpredictable rainfall patterns, as suggested by some studies (Procheş, Cowling, Goldblatt, Manning, & Snijman, 2006; Procheş et al., 2005), previous work (Howard et al., 2019), as well as this study, elevates the potentially greater importance of temperature to geophyte evolution since variables related to precipitation were either not found to be significant (this study) or were significant but to a lesser extent compared to temperature (Howard et al., 2019).

Our results showing a correlation between bulb size and thermal variables brings us to generate hypotheses requiring further testing. For example, larger plants have greater photosynthetic capacity as shown in some epiphytes (Testo & Watkins, 2012; Zotz, 1997). In plants with belowground stores, USO size has a positive correlation with leaf biomass as well as flowering reliability and quality (De Mastro & Ruta, 1993; Han, 2001; Hanzawa & Kalisz, 1993; Klimešová et al., 2017; Rees, 1969). Taking these studies into account, we hypothesize that in order to replenish and maintain larger bulbs without hampering other physiological or life‐history processes, hysteranthous taxa may have greater leaf surface area and photosynthetic capacity relative to synanthous taxa. Additionally, these larger USOs may allow for bigger, more numerous‐flowered inflorescences.

Hysteranthous taxa have larger overall bulbs compared to synanthous taxa (Figure 4). Anecdotally, a similar pattern has been noted in tuberous, hysteranthous *Cyclamen* when compared to synanthous relatives (Debussche, Garnier, & Thompson, 2004). Adequate USO size is likely a stronger prerequisite for flowering in hysteranthous plants than synanthous taxa since these USOs power flower emergence typically during the dry season and without leaves present, thus, leaving hysteranthous taxa to rely on larger reserves to fuel this process (Dafni, Cohen, et al., 1981; Dafni, Shmida, et al., 1981; Rees, 1972; Ruiters, McKenzie, & Raitt, 1993). In the Mediterranean basin, it has been hypothesized that flowering outside of the main flowering season (i.e., the wet season) may be advantageous due to reduced competition for pollinators (Dafni, 1996; Dafni, Cohen, et al., 1981; Dafni, Shmida, et al., 1981; Ruiters et al., 1993). Perennial, hysteranthous taxa typically flower annually once a certain USO size has been obtained, sometimes without the appearance of leaves for consecutive years (Dafni, Cohen, et al., 1981). These factors increase chances for reproductive success, which may have driven selection to act upon populations that flower earlier, ultimately leading to increases in overall bulb size to support this flowering consistency in hysteranthous taxa.

### 4.1 Future directions

The separation of flower and leaf emergence in hysteranthous taxa, and the temporal coupling of flower and leaf emergence in synanthous taxa are sometimes expressed along a spectrum, rather than in defined, predictable stages (Dafni, Cohen, et al., 1981; Dafni, Shmida, et al., 1981; Debussche et al., 2004; Marques & Draper, 2012). For example, when grown in consistently wet conditions, hysteranthous *Pancratium maritimum* can adopt a synanthous phenology (Dafni, Cohen, et al., 1981). Additionally, synanthous taxa show varying degrees of leaf emergence at the time of flowering with leaves either slightly, partly, or fully emerged (Debussche et al., 2004). Future research should quantify and investigate the significance of these spectra in order to understand the degree to which it is expressed in specific ecological settings and the ensuing effect on USO size. Additionally, studies on hysteranthous taxa should also consider whether flowers are borne prior to or after the annual leafing cycle (i.e., *Crocus* vs. *Urginea* type; Dafni, Cohen, et al., 1981).

In this study, we focused only on the putative significance of tunicate bulb size in relation to phenology and ecology. In addition to USO size, it is likely worthwhile to also incorporate other morphological aspects of the plant body into future studies, such as overall plant height, stem size (i.e., the basal plate), leaf number and size, and inflorescence size and number of flowers. Future work should also consider annual versus perennial bulbs, vegetative propagation potential (i.e., clonal offsets) as well as depth below the soil line. Controlling for these other aspects may highlight interesting tradeoffs between resource sources and sinks, and how they relate to the evolution of size variation in bulbs as well as other USOs.

Collection‐based studies, regardless of their limitations, are critical to generate and test broad, compelling evolutionary hypotheses that include a comprehensive diversity of taxa otherwise not easily accessible. The use of herbarium specimens is not without challenges though since suitable bulbs need to be carefully selected because of potential crushing and shrinking due to the normal specimen preservation process. In addition to larger bulbs being less represented in collections, we also observed that many specimens are simply missing their belowground structures. Therefore, when possible, the above recommendations should be carried out using field‐based and experimental set‐ups using living specimens in order to capture important morphological and ecological components that will allow for a more detailed assessment of the processes controlling USO size as well as its downstream effects on plant growth.

## 5 CONCLUSIONS

Capitalizing on the availability of museum collections, we set out to understand some of the potential ecological factors associated with bulb size in monocots. Interpreting our results within a phylogenetic framework, we found that temperature is likely a greater constraint on USO size than precipitation. Our results also support the hypothesis that hysteranthous taxa possess larger bulbs relative to synanthous taxa. This study further highlights the importance of incorporating belowground traits into plant studies, as well as the need for greater representation of USOs in museum collections. More broadly, this work contributes to our growing understanding of the ecological consequences associated with size changes in plants with different growth habits. Future work should be carried out at different geographic and phylogenetic scales in order to gain a deeper understanding of the ecological and evolutionary history of USO size. This study represents a first step toward that goal.

## CONFLICT OF INTEREST

None declared.

## AUTHOR CONTRIBUTIONS

CCH conceived the project, collected data, ran analyses, and wrote manuscript; NC improved project design, helped with interpretation of results and with writing the manuscript.

## Data Availability

GBIF identification numbers and download information can be found in Supporting Document 1. All data used in analyses can be found in Digital Dryad (https://doi.org/10.5061/dryad.sf7m0cg25).
