# Supplementary material for: Tunicate bulb size variation in monocots explained by temperature and phenology
Source: Ecol Evol. 2020 Feb 27;10(5):2299–309. doi: 10.1002/ece3.5996 (PMC7069286; doi:10.1002/ece3.5996)
Supplement: Supplementary file 1 [file ECE3-10-2299-s001.docx]

Taxon selection and corresponding GBIF (www.gbif.org) download information.

*Acis autumnalis*

GBIF.org (31st August 2018) GBIF Occurrence Download <https://urldefense.proofpoint.com/v2/url?u=https-3A__doi.org_10.15468_dl.mnoeie&d=DwICAg&c=pZJPUDQ3SB9JplYbifm4nt2lEVG5pWx2KikqINpWlZM&r=9iE1-ZuRcrK1bcpQgDReSQ&m=lgmMkKn9GuFxiEWuqyPfmGZEWdoaXM1rBdh1ErL4Bu0&s=otZNCaRxIwufinm4NCkvDXUgzxQQCf1icSUsUJqxqBQ&e=>

*Albuca abyssinica*

GBIF.org (31st August 2018) GBIF Occurrence Download <https://urldefense.proofpoint.com/v2/url?u=https-3A__doi.org_10.15468_dl.exo4h7&d=DwICAg&c=pZJPUDQ3SB9JplYbifm4nt2lEVG5pWx2KikqINpWlZM&r=9iE1-ZuRcrK1bcpQgDReSQ&m=WGK9GPFyJ8FQOs1qOX5s2QpHbYOTq2FqareDXEG14B4&s=OLH_33EVWgCO89SpJeZAVp_8bJmJZnn3s4mpZao9HKs&e=>

*Allium borszczowii*

GBIF.org (31st May 2018) GBIF Occurrence Download <https://urldefense.proofpoint.com/v2/url?u=https-3A__doi.org_10.15468_dl.keures&d=DwICAg&c=pZJPUDQ3SB9JplYbifm4nt2lEVG5pWx2KikqINpWlZM&r=9iE1-ZuRcrK1bcpQgDReSQ&m=k8e305XLDrMGi3ZHutwS-qfGdiqwbTlbuyv0LL8vlH4&s=yRPkpPQ-VcdFcBcSzvrWNyh_XO2KPKQZaK4U-Uc41rM&e=>

*Allium chinense*

GBIF.org (31st May 2018) GBIF Occurrence Download <https://urldefense.proofpoint.com/v2/url?u=https-3A__doi.org_10.15468_dl.rf6u5d&d=DwICAg&c=pZJPUDQ3SB9JplYbifm4nt2lEVG5pWx2KikqINpWlZM&r=9iE1-ZuRcrK1bcpQgDReSQ&m=fhI6xvuHNYEP0SAVjStkbvTYh_gHE0uLbKKKvBHzDo0&s=wmRMgNJBNlTkmOgkgzoRJGdx1jMWAbj9nFgJSKuMLd0&e=>

*Allium moly*

GBIF.org (31st May 2018) GBIF Occurrence Download <https://urldefense.proofpoint.com/v2/url?u=https-3A__doi.org_10.15468_dl.wskjwc&d=DwICAg&c=pZJPUDQ3SB9JplYbifm4nt2lEVG5pWx2KikqINpWlZM&r=9iE1-ZuRcrK1bcpQgDReSQ&m=vPAPrINZqg-JA-N0KmAL28ae0T-S-RKcuO2zjpOWNIs&s=PFuBfvAkQNTdBHAXrcScvRBbeoNnLQktysuPqdFR9ZY&e=>

*Allium pallasii*

GBIF.org (31st May 2018) GBIF Occurrence Download <https://urldefense.proofpoint.com/v2/url?u=https-3A__doi.org_10.15468_dl.uiqffb&d=DwICAg&c=pZJPUDQ3SB9JplYbifm4nt2lEVG5pWx2KikqINpWlZM&r=9iE1-ZuRcrK1bcpQgDReSQ&m=sxgkk-pgOTXQUW_KolShld70ES2OdzNot1GD7yjyDaQ&s=a-yrdYjcOZDmpVBu2_MfzzJdF4pvrPs6OqV3Fn-3JSA&e=>

*Amana edulis*

GBIF.org (31st August 2018) GBIF Occurrence Download <https://urldefense.proofpoint.com/v2/url?u=https-3A__doi.org_10.15468_dl.cdukty&d=DwICAg&c=pZJPUDQ3SB9JplYbifm4nt2lEVG5pWx2KikqINpWlZM&r=9iE1-ZuRcrK1bcpQgDReSQ&m=m67Co-PFJzhMISLmz-SkmCotZuN3wpryiRbhKJRrlcQ&s=ACyLpbkEU9awQTeWrIygNvVjid8r961wlSP4lf8cIwY&e=>

*Amaryllis belladonna*

GBIF.org (31st August 2018) GBIF Occurrence Download <https://urldefense.proofpoint.com/v2/url?u=https-3A__doi.org_10.15468_dl.bvdggt&d=DwICAg&c=pZJPUDQ3SB9JplYbifm4nt2lEVG5pWx2KikqINpWlZM&r=9iE1-ZuRcrK1bcpQgDReSQ&m=k84nfryoybanfJ6n4C7908GOLlmYU42W5u09G9drtUs&s=1_ZyeIpKmx1FGHn83LbWnWnP0sRW9oKAuHL379sM1hg&e=>

*Ammocharis tinneana*

GBIF.org (31st August 2018) GBIF Occurrence Download <https://urldefense.proofpoint.com/v2/url?u=https-3A__doi.org_10.15468_dl.rigz6f&d=DwICAg&c=pZJPUDQ3SB9JplYbifm4nt2lEVG5pWx2KikqINpWlZM&r=9iE1-ZuRcrK1bcpQgDReSQ&m=336JWamrNhmK7rLxethB8-Bli-_YDhwSb7o9vOOIjQc&s=1X2Z92kpJQpp9GlpGBi9Zk7LqkJCzZRvpP8zugNuLgw&e=>

*Bellevalia romana*

GBIF.org (31st August 2018) GBIF Occurrence Download <https://urldefense.proofpoint.com/v2/url?u=https-3A__doi.org_10.15468_dl.2nuqqe&d=DwICAg&c=pZJPUDQ3SB9JplYbifm4nt2lEVG5pWx2KikqINpWlZM&r=9iE1-ZuRcrK1bcpQgDReSQ&m=bir4r56FL7XdQ0TZUAvrCtREogbBpxnKZaMaR633GZ4&s=K19SXrtyYIzfExPQ6wRvrvXCPucQA18qQ_CP3pfVq30&e=>

*Calochortus barbatus*

GBIF.org (29th May 2018) GBIF Occurrence Download <https://urldefense.proofpoint.com/v2/url?u=https-3A__doi.org_10.15468_dl.ulqwpz&d=DwICAg&c=pZJPUDQ3SB9JplYbifm4nt2lEVG5pWx2KikqINpWlZM&r=9iE1-ZuRcrK1bcpQgDReSQ&m=mIGNPwTwkU0iAZBnTJnCiO14HB0-y_cIa1_frXD7UaU&s=LJMLhWKZZyphWKfIFaMBubUaqQics8-wBbZT4-3fhyA&e=>

*Calochortus gunnisonii*

GBIF.org (21st August 2018) GBIF Occurrence Download <https://urldefense.proofpoint.com/v2/url?u=https-3A__doi.org_10.15468_dl.cqurky&d=DwICAg&c=pZJPUDQ3SB9JplYbifm4nt2lEVG5pWx2KikqINpWlZM&r=9iE1-ZuRcrK1bcpQgDReSQ&m=BaIbguBrVY03QUTsAIZ6n20fcdlUyebIvah5HrriNdc&s=Egj29eZl2l49BVJqBDI0EXUpwAYt5dE6_-DArM2Sz5o&e=>

*Calochortus kennedyi*

GBIF.org (31st August 2018) GBIF Occurrence Download <https://urldefense.proofpoint.com/v2/url?u=https-3A__doi.org_10.15468_dl.fzwzou&d=DwICAg&c=pZJPUDQ3SB9JplYbifm4nt2lEVG5pWx2KikqINpWlZM&r=9iE1-ZuRcrK1bcpQgDReSQ&m=aPyKzh8K4pbHSG6ynCI3ROhGi4Usq-SC-OupS1PjVTM&s=goxP4T7zXSCve9NManWeast2s7Jqm5og4eoXWVZ3lWI&e=>

*Camassia quamash*

GBIF.org (31st August 2018) GBIF Occurrence Download <https://urldefense.proofpoint.com/v2/url?u=https-3A__doi.org_10.15468_dl.anbpdl&d=DwICAg&c=pZJPUDQ3SB9JplYbifm4nt2lEVG5pWx2KikqINpWlZM&r=9iE1-ZuRcrK1bcpQgDReSQ&m=2V7gSEk7FEfA9gBRbrAgCKBx5pP5KA5BOjPVP9fTYVk&s=sQiG91QRa1XuhvObU8Ro3qPmNZfOEZZG4voAARu47G4&e=>

*Camassia scilloides*

GBIF.org (31st August 2018) GBIF Occurrence Download <https://urldefense.proofpoint.com/v2/url?u=https-3A__doi.org_10.15468_dl.fxxbvw&d=DwICAg&c=pZJPUDQ3SB9JplYbifm4nt2lEVG5pWx2KikqINpWlZM&r=9iE1-ZuRcrK1bcpQgDReSQ&m=bwLm9hM34Mmlkun50mrUJbtLQXUPfqev6K5GvQL-aGg&s=xz_dhDa-vle_E253NIcFseND56_-uj8vhLb8Cmi7P-w&e=>

*Clinanthus croceus*

GBIF.org (31st August 2018) GBIF Occurrence Download <https://urldefense.proofpoint.com/v2/url?u=https-3A__doi.org_10.15468_dl.kcqiw5&d=DwICAg&c=pZJPUDQ3SB9JplYbifm4nt2lEVG5pWx2KikqINpWlZM&r=9iE1-ZuRcrK1bcpQgDReSQ&m=XjE73Lup4JYDPuUropAQZGS4CRRK1GX4pDBlQb4OlK0&s=FLakhmxUh1zItqFyOzKZtoMutkNGd0_2eoVqqujl9kE&e=>

*Crinum natans*

GBIF.org (31st May 2018) GBIF Occurrence Download <https://urldefense.proofpoint.com/v2/url?u=https-3A__doi.org_10.15468_dl.gqmwjt&d=DwICAg&c=pZJPUDQ3SB9JplYbifm4nt2lEVG5pWx2KikqINpWlZM&r=9iE1-ZuRcrK1bcpQgDReSQ&m=zRvOHF3xkPtLZY1b8tXXv3b15x1ytXNv5z7uoEELWsg&s=H87Pw7zyFVm77jo6W-Vqb7NGeY-kyINDuql3mEwanlQ&e=>

*Crinum zeylanicum*

GBIF.org (28th August 2018) GBIF Occurrence Download <https://urldefense.proofpoint.com/v2/url?u=https-3A__doi.org_10.15468_dl.upotrx&d=DwICAg&c=pZJPUDQ3SB9JplYbifm4nt2lEVG5pWx2KikqINpWlZM&r=9iE1-ZuRcrK1bcpQgDReSQ&m=7rPZb9jxpIrZsiRJQde75qJpiAYzHu8novpBcVBfeOA&s=GE7EoTC1pdcXCd-OJs-5EQw1RAOhPI2lNOBRAWRDRFA&e=>

*Cyrtanthus breviflorus*

GBIF.org (28th August 2018) GBIF Occurrence Download <https://urldefense.proofpoint.com/v2/url?u=https-3A__doi.org_10.15468_dl.hsm5tz&d=DwICAg&c=pZJPUDQ3SB9JplYbifm4nt2lEVG5pWx2KikqINpWlZM&r=9iE1-ZuRcrK1bcpQgDReSQ&m=zEoJvUMW9qPeE0UwMzSSOtLQIc4krS3Nvhd1ekQO2-0&s=xz9XnJJkY2jqiLg7hksUFXvZ3gasV3m0Zyr9iq3Qd2E&e=>

*Cyrtanthus sanguineus*

GBIF.org (28th August 2018) GBIF Occurrence Download <https://urldefense.proofpoint.com/v2/url?u=https-3A__doi.org_10.15468_dl.yape59&d=DwICAg&c=pZJPUDQ3SB9JplYbifm4nt2lEVG5pWx2KikqINpWlZM&r=9iE1-ZuRcrK1bcpQgDReSQ&m=2lTgjjk6T5rnM2EqPuZ1bvRhXzZv1HfLJUd4ESJwZv0&s=7zN5ewSzoswh9X3h3Pzp8KHUUNlVBAimi5wPN0bXroA&e=>

*Dipcadi longifolium*

GBIF.org (31st May 2018) GBIF Occurrence Download <https://urldefense.proofpoint.com/v2/url?u=https-3A__doi.org_10.15468_dl.wfoxho&d=DwICAg&c=pZJPUDQ3SB9JplYbifm4nt2lEVG5pWx2KikqINpWlZM&r=9iE1-ZuRcrK1bcpQgDReSQ&m=Xa1raCsI9OaYLyLbtffEh-P0iffpj6i38gFyluNsF90&s=UyzhLOaeaeMVg9Qa3YUmVpCwWKnF8SLM8h0UKJgBn_k&e=>

*Dipcadi serotinum*

GBIF.org (31st May 2018) GBIF Occurrence Download <https://urldefense.proofpoint.com/v2/url?u=https-3A__doi.org_10.15468_dl.00cd8k&d=DwICAg&c=pZJPUDQ3SB9JplYbifm4nt2lEVG5pWx2KikqINpWlZM&r=9iE1-ZuRcrK1bcpQgDReSQ&m=02qU8DQBi8REt5bqd8Z_hE5JlMq3eSDaDr1xoPEW59M&s=wxGhZNgOA4X3lOF1y3-fSTqiTH83kaUS9wDLq_F5jJE&e=>

*Dipcadi viride*

GBIF.org (31st May 2018) GBIF Occurrence Download <https://urldefense.proofpoint.com/v2/url?u=https-3A__doi.org_10.15468_dl.g8aa6x&d=DwICAg&c=pZJPUDQ3SB9JplYbifm4nt2lEVG5pWx2KikqINpWlZM&r=9iE1-ZuRcrK1bcpQgDReSQ&m=QyRHT8WpecWFL7ITBb7tBhemTGS-zrGay5f2aEI2WAY&s=mj-4BBwVSJRiFmwQwErOB0RxA8r1tb-rlCTLH8jfJPo&e=>

*Drimia indica*

GBIF.org (31st May 2018) GBIF Occurrence Download <https://urldefense.proofpoint.com/v2/url?u=https-3A__doi.org_10.15468_dl.k38gom&d=DwICAg&c=pZJPUDQ3SB9JplYbifm4nt2lEVG5pWx2KikqINpWlZM&r=9iE1-ZuRcrK1bcpQgDReSQ&m=K9rRDRFdocJukusmQUEl79GZSJqAamAjTHgQ9vPg95E&s=5J9xOa7iL1VKVHnGYdHlx85IQ3Ca-Cdeu7XMAJVhafc&e=>

*Drimia mascarenensis*

GBIF.org (31st May 2018) GBIF Occurrence Download <https://urldefense.proofpoint.com/v2/url?u=https-3A__doi.org_10.15468_dl.rjd9mv&d=DwICAg&c=pZJPUDQ3SB9JplYbifm4nt2lEVG5pWx2KikqINpWlZM&r=9iE1-ZuRcrK1bcpQgDReSQ&m=rO-qK6hYVv6M_mBhDY0WmOzZn3gysH5F19e89c-oCjo&s=_IgPuA0UoKBPKGKuT65R94PBQqrzM80OAydyAkoctek&e=>

*Drimia noctiflora*

GBIF.org (31st May 2018) GBIF Occurrence Download <https://urldefense.proofpoint.com/v2/url?u=https-3A__doi.org_10.15468_dl.sgey2e&d=DwICAg&c=pZJPUDQ3SB9JplYbifm4nt2lEVG5pWx2KikqINpWlZM&r=9iE1-ZuRcrK1bcpQgDReSQ&m=89Vg8XH0RRTq-7HZj-PmVL9T6SDrZrytV6gELaqEexc&s=lF3oqarXwTpGgMyusnHJlLn5p-2iOjpteG7vvkn-3pc&e=>

*Drimiopsis barteri*

GBIF.org (29th May 2018) GBIF Occurrence Download <https://urldefense.proofpoint.com/v2/url?u=https-3A__doi.org_10.15468_dl.ni3qjo&d=DwICAg&c=pZJPUDQ3SB9JplYbifm4nt2lEVG5pWx2KikqINpWlZM&r=9iE1-ZuRcrK1bcpQgDReSQ&m=OYhH_hq7-25py8PF5-vBvCO7ozxDzS1OzaeHn0lIRo0&s=dC51qBPl5e6RpCyJG98nfUHPXKugaCOL52-9YmZv8yE&e=>

*Drimiopsis botryoides*

GBIF.org (29th May 2018) GBIF Occurrence Download <https://urldefense.proofpoint.com/v2/url?u=https-3A__doi.org_10.15468_dl.hbiygj&d=DwICAg&c=pZJPUDQ3SB9JplYbifm4nt2lEVG5pWx2KikqINpWlZM&r=9iE1-ZuRcrK1bcpQgDReSQ&m=W-WhnNRcBXiZKJPUyYt1_0iLkuKHV62BqnSAjFMzpYw&s=1UPhk2wnFijIa-r8romnJm-C9r138bLcvMPdAe29MFI&e=>

*Drimiopsis maculata*

GBIF.org (29th May 2018) GBIF Occurrence Download <https://urldefense.proofpoint.com/v2/url?u=https-3A__doi.org_10.15468_dl.bbixgx&d=DwICAg&c=pZJPUDQ3SB9JplYbifm4nt2lEVG5pWx2KikqINpWlZM&r=9iE1-ZuRcrK1bcpQgDReSQ&m=CkEZcevShB7gfR3H7iuhcVfWuTLihSEZZc3Rkahar5I&s=vPAWNzprRpJV9YmAwV-zlupxBsueho5oLMlG3JEQQi4&e=>

*Galanthus alpinus*

GBIF.org (31st May 2018) GBIF Occurrence Download <https://urldefense.proofpoint.com/v2/url?u=https-3A__doi.org_10.15468_dl.oyr6db&d=DwICAg&c=pZJPUDQ3SB9JplYbifm4nt2lEVG5pWx2KikqINpWlZM&r=9iE1-ZuRcrK1bcpQgDReSQ&m=todHv3oTykmLORIEXougZqKF6E6ZffY08l1Cfe2B6pI&s=xds74TDDUEiUugA1PnyzCZXpuM2OofQDsi3JS0nQ9o4&e=>

*Galanthus elwesii*

GBIF.org (31st May 2018) GBIF Occurrence Download <https://urldefense.proofpoint.com/v2/url?u=https-3A__doi.org_10.15468_dl.3vkwal&d=DwICAg&c=pZJPUDQ3SB9JplYbifm4nt2lEVG5pWx2KikqINpWlZM&r=9iE1-ZuRcrK1bcpQgDReSQ&m=zBLooF5mLeK4kYhbin3XF451_bIbKyLO_mpDwCYw9Ac&s=HKKCb-jQJhoP_AeMCxie9XDQtSpfxamrsYxa1QaLESU&e=>

*Gethyllis spiralis*

GBIF.org (31st August 2018) GBIF Occurrence Download <https://urldefense.proofpoint.com/v2/url?u=https-3A__doi.org_10.15468_dl.omoz8o&d=DwICAg&c=pZJPUDQ3SB9JplYbifm4nt2lEVG5pWx2KikqINpWlZM&r=9iE1-ZuRcrK1bcpQgDReSQ&m=XS12UCn-ORAm9WqHmpx8OLihxS_UtfZkqBH0I_YZEwk&s=-qjcDHyX4UknRt_lbSFt7ICYRPFCXS6ktzUssYJBASE&e=>

*Habranthus longifolius*

GBIF.org (31st May 2018) GBIF Occurrence Download <https://urldefense.proofpoint.com/v2/url?u=https-3A__doi.org_10.15468_dl.y6aqqf&d=DwICAg&c=pZJPUDQ3SB9JplYbifm4nt2lEVG5pWx2KikqINpWlZM&r=9iE1-ZuRcrK1bcpQgDReSQ&m=wEhgLRvJTO9fVnJco1KgJMRxOkuysh_v0XqO8_xXxvI&s=3TDUOAZXakr07VOE1FUDhbP3ZuQQ3tj539aQY0Cxa1E&e=>

*Hesperocallis undulata*

GBIF.org (4th June 2018) GBIF Occurrence Download <https://urldefense.proofpoint.com/v2/url?u=https-3A__doi.org_10.15468_dl.j31opn&d=DwICAg&c=pZJPUDQ3SB9JplYbifm4nt2lEVG5pWx2KikqINpWlZM&r=9iE1-ZuRcrK1bcpQgDReSQ&m=-WsrM4pZmjvZGb3R2B97vb-ZcZcdX_YY-_ygtotW_M4&s=uJH1ZQ5-is6Wd3a8b-FM2BPDjNn5eskKiEeVgZz10A0&e=>

*Hessea cinnamomea*

GBIF.org (31st August 2018) GBIF Occurrence Download <https://urldefense.proofpoint.com/v2/url?u=https-3A__doi.org_10.15468_dl.hmu0wy&d=DwICAg&c=pZJPUDQ3SB9JplYbifm4nt2lEVG5pWx2KikqINpWlZM&r=9iE1-ZuRcrK1bcpQgDReSQ&m=UdIyABsQGSqNa7IIWVaegNInt6G76bNW2RdGYTVF4MU&s=WGD-AIJftFsWJ60aK2cljO16YVgYSifLLHshkorrRLM&e=>

*Hippeastrum aulicum*

GBIF.org (31st May 2018) GBIF Occurrence Download <https://urldefense.proofpoint.com/v2/url?u=https-3A__doi.org_10.15468_dl.qubll8&d=DwICAg&c=pZJPUDQ3SB9JplYbifm4nt2lEVG5pWx2KikqINpWlZM&r=9iE1-ZuRcrK1bcpQgDReSQ&m=x4lqiZpQ4fz0rRmK7C62MBIrk_hj4NkkOvkg8MydMVM&s=2hXFWB7-t14Ql9tS1miAeKO7kXY2mqlycjUS6ejRfcM&e=>

*Hippeastrum puniceum*

GBIF.org (31st May 2018) GBIF Occurrence Download <https://urldefense.proofpoint.com/v2/url?u=https-3A__doi.org_10.15468_dl.z7ghnr&d=DwICAg&c=pZJPUDQ3SB9JplYbifm4nt2lEVG5pWx2KikqINpWlZM&r=9iE1-ZuRcrK1bcpQgDReSQ&m=Wldz0LzYY654SuAaEcuPSU0RH82l3tULORzKxQJrEpY&s=jfLrg_nYYmcYAsmC7_j-xaky1Bx80Qgww_-XpISJKHo&e=>

*Hippeastrum reginae*

GBIF.org (31st May 2018) GBIF Occurrence Download <https://urldefense.proofpoint.com/v2/url?u=https-3A__doi.org_10.15468_dl.k3pbdf&d=DwICAg&c=pZJPUDQ3SB9JplYbifm4nt2lEVG5pWx2KikqINpWlZM&r=9iE1-ZuRcrK1bcpQgDReSQ&m=oJPWqeYzC5gG4AMko9hV0sw7xsmF1EMzLsAmgiBuWXo&s=7a7kYjQ0vszhwoCYMEA3fXPZ4fr1W_fXnXdiGxl7qWI&e=>

*Hyacinthoides aristidis*

GBIF.org (31st May 2018) GBIF Occurrence Download <https://urldefense.proofpoint.com/v2/url?u=https-3A__doi.org_10.15468_dl.b3eger&d=DwICAg&c=pZJPUDQ3SB9JplYbifm4nt2lEVG5pWx2KikqINpWlZM&r=9iE1-ZuRcrK1bcpQgDReSQ&m=Yjqkee0ZO-eHI0pgheE9a9d4ESI75rVEW7HOTJJgNT4&s=82sSHLG-x9rvfnPp9F-lOx9H_zqZABU4-1YvjYhQSGQ&e=>

*Hyacinthoides hispanica*

GBIF.org (31st May 2018) GBIF Occurrence Download <https://urldefense.proofpoint.com/v2/url?u=https-3A__doi.org_10.15468_dl.ojmftf&d=DwICAg&c=pZJPUDQ3SB9JplYbifm4nt2lEVG5pWx2KikqINpWlZM&r=9iE1-ZuRcrK1bcpQgDReSQ&m=wySDFSSHEJ81XNx9A1GDhbtv3lV-Dfc5tDUIK2tdzvE&s=7Fzeo7S14Fq7kCtfCsMbKL_AXOlCTV9ritIna5zXATo&e=>

*Hyacinthoides non-scripta*

GBIF.org (31st May 2018) GBIF Occurrence Download <https://urldefense.proofpoint.com/v2/url?u=https-3A__doi.org_10.15468_dl.3f8ujg&d=DwICAg&c=pZJPUDQ3SB9JplYbifm4nt2lEVG5pWx2KikqINpWlZM&r=9iE1-ZuRcrK1bcpQgDReSQ&m=P3AUIOMyXLo0BzyEF4HqzzfLOLeo29aDds8_nMuYC10&s=ChT-LmF2OE0HLA0b2rZzUoQ6dsSekqsuttv7B9El2qs&e=>

*Hyacinthus litwinowii*

GBIF.org (31st May 2018) GBIF Occurrence Download <https://urldefense.proofpoint.com/v2/url?u=https-3A__doi.org_10.15468_dl.08ggua&d=DwICAg&c=pZJPUDQ3SB9JplYbifm4nt2lEVG5pWx2KikqINpWlZM&r=9iE1-ZuRcrK1bcpQgDReSQ&m=NXuPgSWGoK7vefrRg7ZD42PlIPjHB9PvSo1u3lZkf4U&s=I6XTnS5YRmwcVHXdYRyZ1gGpZhcFfpCTDmXeqohah6U&e=>

*Ipheion uniflorum*

GBIF.org (31st August 2018) GBIF Occurrence Download <https://urldefense.proofpoint.com/v2/url?u=https-3A__doi.org_10.15468_dl.cafvy0&d=DwICAg&c=pZJPUDQ3SB9JplYbifm4nt2lEVG5pWx2KikqINpWlZM&r=9iE1-ZuRcrK1bcpQgDReSQ&m=ROETXyhaKr4ixfrlQ5I6a8PjFIZWiEqstlR-mfgSCHI&s=cIMQgzKU6bXxPydud-DFHhxeKhyi6txMHZNTddHcB5k&e=>

*Iris danfordiae*

GBIF.org (31st May 2018) GBIF Occurrence Download <https://urldefense.proofpoint.com/v2/url?u=https-3A__doi.org_10.15468_dl.jzdw0z&d=DwICAg&c=pZJPUDQ3SB9JplYbifm4nt2lEVG5pWx2KikqINpWlZM&r=9iE1-ZuRcrK1bcpQgDReSQ&m=HK4nUYyqShEaHqat4vuuMLJh6900-RoEdqWGurIdjmY&s=F7AJ8oUuoR2DqsVs4TTA_ycbw9HdzWEK3-5WkF4vcho&e=>

*Iris kolpakowskiana*

GBIF.org (31st May 2018) GBIF Occurrence Download <https://urldefense.proofpoint.com/v2/url?u=https-3A__doi.org_10.15468_dl.0uysys&d=DwICAg&c=pZJPUDQ3SB9JplYbifm4nt2lEVG5pWx2KikqINpWlZM&r=9iE1-ZuRcrK1bcpQgDReSQ&m=tT-Gg6tpuwpQ6rF0HAocjaOX-VSZY82DIUW-dxB4VC4&s=FvbkbiuNxgb15boGu-FuE8BLmPlrKSia0VbgeAv2WiI&e=>

*Iris persica*

GBIF.org (31st May 2018) GBIF Occurrence Download <https://urldefense.proofpoint.com/v2/url?u=https-3A__doi.org_10.15468_dl.f3imqb&d=DwICAg&c=pZJPUDQ3SB9JplYbifm4nt2lEVG5pWx2KikqINpWlZM&r=9iE1-ZuRcrK1bcpQgDReSQ&m=dxriuOy04er36q7pRBiTIymSdN2MNEH1BF2lZdDd6KE&s=IUy89WJtNpldPfiKgQC_84k7URIUVLWpotP2xup6nu0&e=>

*Iris reticulata*

GBIF.org (27th September 2018) GBIF Occurrence Download <https://urldefense.proofpoint.com/v2/url?u=https-3A__doi.org_10.15468_dl.ioy95l&d=DwICAg&c=pZJPUDQ3SB9JplYbifm4nt2lEVG5pWx2KikqINpWlZM&r=9iE1-ZuRcrK1bcpQgDReSQ&m=C9XV4CuPhcrztfskY6HWyYM2lRsfrVUOQb0EEPiEJvQ&s=KqTVYDzyTTXujDm7JTWlvifWRfOMx4L71UiqvLLe2aY&e=>

*Iris xiphium*

GBIF.org (31st August 2018) GBIF Occurrence Download <https://urldefense.proofpoint.com/v2/url?u=https-3A__doi.org_10.15468_dl.pa6kc9&d=DwICAg&c=pZJPUDQ3SB9JplYbifm4nt2lEVG5pWx2KikqINpWlZM&r=9iE1-ZuRcrK1bcpQgDReSQ&m=45SkOxdAqT6OL7zF3qa4kxGnSRUdJRTAAdUje3-87_s&s=zDdUB88NwhX2phaI7ot5xnf_LCO2HWOB0blauNZizzU&e=>

*Ismene amancaes*

GBIF.org (31st May 2018) GBIF Occurrence Download <https://urldefense.proofpoint.com/v2/url?u=https-3A__doi.org_10.15468_dl.qa1ndg&d=DwICAg&c=pZJPUDQ3SB9JplYbifm4nt2lEVG5pWx2KikqINpWlZM&r=9iE1-ZuRcrK1bcpQgDReSQ&m=yKBHXOXaUH54OHruMTD4v2o_v5oSvhBk3nzXPd9PtUw&s=AatSM-XGpw67e59Bzp260uhksXGjpUjm96ChFJNt54k&e=>

*Lachenalia contaminata*

GBIF.org (29th May 2018) GBIF Occurrence Download <https://urldefense.proofpoint.com/v2/url?u=https-3A__doi.org_10.15468_dl.2b6lev&d=DwICAg&c=pZJPUDQ3SB9JplYbifm4nt2lEVG5pWx2KikqINpWlZM&r=9iE1-ZuRcrK1bcpQgDReSQ&m=DbUcyqlXwTBFggziN5iDGA5hkoSE-M7oX9Fxd7pjGR4&s=DC3b0DubxYh9162aVp7Zu-1EWgjcFKPMNAgN2LLQEBs&e=>

*Lachenalia corymbosa*

GBIF.org (29th May 2018) GBIF Occurrence Download <https://urldefense.proofpoint.com/v2/url?u=https-3A__doi.org_10.15468_dl.yev22y&d=DwICAg&c=pZJPUDQ3SB9JplYbifm4nt2lEVG5pWx2KikqINpWlZM&r=9iE1-ZuRcrK1bcpQgDReSQ&m=DuPUq83kcesDGlh_YTpvgXUaV7RC43whbWzQ_Tc09tw&s=uqM3STBk4ZKE8Lybf3a1rm6q3qmNYNF7kUa3fqqXOa4&e=>

*Lachenalia orchioides*

GBIF.org (29th May 2018) GBIF Occurrence Download <https://urldefense.proofpoint.com/v2/url?u=https-3A__doi.org_10.15468_dl.xo4tap&d=DwICAg&c=pZJPUDQ3SB9JplYbifm4nt2lEVG5pWx2KikqINpWlZM&r=9iE1-ZuRcrK1bcpQgDReSQ&m=RW0YYXihNU2iNbegdH6LhuGarUyvCc9gQWN0HKYjvgY&s=2cxHUGUdONCIUYCFpDqhYYZApWbiiSUFu3E92xTBVwY&e=>

*Ledebouria camerooniana*

GBIF.org (29th May 2018) GBIF Occurrence Download <https://urldefense.proofpoint.com/v2/url?u=https-3A__doi.org_10.15468_dl.1exdkl&d=DwICAg&c=pZJPUDQ3SB9JplYbifm4nt2lEVG5pWx2KikqINpWlZM&r=9iE1-ZuRcrK1bcpQgDReSQ&m=Uu0tAfTqOs-3s8KM-LxNuv7YX_8RRgRGwbH-zoZcSt8&s=oTkF3xYF9K7Tdk2HMJ0Ff29awVCzVxhuBGvqW5EEP9o&e=>

*Ledebouria cooperi*

GBIF.org (29th May 2018) GBIF Occurrence Download <https://urldefense.proofpoint.com/v2/url?u=https-3A__doi.org_10.15468_dl.7ahte4&d=DwICAg&c=pZJPUDQ3SB9JplYbifm4nt2lEVG5pWx2KikqINpWlZM&r=9iE1-ZuRcrK1bcpQgDReSQ&m=kIvUf1bXs6EX_B9Pm4RMYwPpFnirc_Mup70Oyig8jek&s=ON4CW4yJ6I-EmbT33EaynX8L_ResawkLjwMZR5cmrgc&e=>

*Ledebouria revoluta*

GBIF.org (29th May 2018) GBIF Occurrence Download <https://urldefense.proofpoint.com/v2/url?u=https-3A__doi.org_10.15468_dl.hizlbm&d=DwICAg&c=pZJPUDQ3SB9JplYbifm4nt2lEVG5pWx2KikqINpWlZM&r=9iE1-ZuRcrK1bcpQgDReSQ&m=KcRmyGifAMKM55x_ooc5VMX19FGxeBfLN0pSmazvsgQ&s=7A6fI04ncPU3j5ay1Xje0GnPR77ypdSnbtvs--IxJjY&e=>

*Ledebouria sudanica*

GBIF.org (29th May 2018) GBIF Occurrence Download <https://urldefense.proofpoint.com/v2/url?u=https-3A__doi.org_10.15468_dl.93rgrq&d=DwICAg&c=pZJPUDQ3SB9JplYbifm4nt2lEVG5pWx2KikqINpWlZM&r=9iE1-ZuRcrK1bcpQgDReSQ&m=dzK8KPq8DLV-7OYL8fGrvx5EclHz9oiWBgCiV2RI69E&s=IoTEjJ3lcNNyL7el9ztc38xBOvrfWfuPy3WCxaiDfxo&e=>

*Leopoldia longipes*

GBIF.org (31st May 2018) GBIF Occurrence Download <https://urldefense.proofpoint.com/v2/url?u=https-3A__doi.org_10.15468_dl.hlwxft&d=DwICAg&c=pZJPUDQ3SB9JplYbifm4nt2lEVG5pWx2KikqINpWlZM&r=9iE1-ZuRcrK1bcpQgDReSQ&m=PIHXBK6EYd-Wny30GZvflQ-0K-TRuFo2VnFWpA9C-P4&s=bGPFNiGozz3-D15s91dwcwT-IqxIYj2j0SSwRZsan6o&e=>

*Leopoldia maritima*

GBIF.org (31st May 2018) GBIF Occurrence Download <https://urldefense.proofpoint.com/v2/url?u=https-3A__doi.org_10.15468_dl.egudox&d=DwICAg&c=pZJPUDQ3SB9JplYbifm4nt2lEVG5pWx2KikqINpWlZM&r=9iE1-ZuRcrK1bcpQgDReSQ&m=pLVcRhg3QS_dL4Te-hVr7hJDlvEkvjU_LnQBMyRK480&s=VG-v_t1hd7nuOP45rUDD2069l1g2Pf4VCNYWf7XgkxU&e=>

*Leopoldia tenuiflora*

GBIF.org (31st May 2018) GBIF Occurrence Download <https://urldefense.proofpoint.com/v2/url?u=https-3A__doi.org_10.15468_dl.7l9qgx&d=DwICAg&c=pZJPUDQ3SB9JplYbifm4nt2lEVG5pWx2KikqINpWlZM&r=9iE1-ZuRcrK1bcpQgDReSQ&m=vVx7PLUrquxb6OusAczRl1yHJ0eChp-6pKiQWoFVaDM&s=eYRQLkJQhOX0hKlFUFMPRUk3kjIaS0L7QDTQCOdY-cs&e=>

*Leucocoryne ixioides*

GBIF.org (31st August 2018) GBIF Occurrence Download <https://urldefense.proofpoint.com/v2/url?u=https-3A__doi.org_10.15468_dl.glu8ja&d=DwICAg&c=pZJPUDQ3SB9JplYbifm4nt2lEVG5pWx2KikqINpWlZM&r=9iE1-ZuRcrK1bcpQgDReSQ&m=HU5KWIL9bkxwKLAPbsB2AlFpOK1aqyWp0uXn0ymm4zw&s=TOaKBLYucxqpQljXFJskpk0F1MOs2RJjZEoid0sqPDU&e=>

*Leucojum vernum*

GBIF.org (31st August 2018) GBIF Occurrence Download <https://urldefense.proofpoint.com/v2/url?u=https-3A__doi.org_10.15468_dl.idnf1x&d=DwICAg&c=pZJPUDQ3SB9JplYbifm4nt2lEVG5pWx2KikqINpWlZM&r=9iE1-ZuRcrK1bcpQgDReSQ&m=6d5Kf5MTJIWlDSWuLr8rwyTMVRGrgUrfq0_jwaDZc2s&s=2aJYFr4Z9s4BFH0RBzzd01Y9henapcBqbUs7RZerKb0&e=>

*Lloydia tibetica*

GBIF.org (31st August 2018) GBIF Occurrence Download <https://urldefense.proofpoint.com/v2/url?u=https-3A__doi.org_10.15468_dl.ga4woi&d=DwICAg&c=pZJPUDQ3SB9JplYbifm4nt2lEVG5pWx2KikqINpWlZM&r=9iE1-ZuRcrK1bcpQgDReSQ&m=2-gcW0GUCuHxbObkwSuq8MDXwnH0Im6r1Tp2ABkVTgI&s=6WSTdVtZApAGmL5jNlQPNjLSFfdlgyyhhCGFrhI8MZg&e=>

*Lycoris aurea*

GBIF.org (31st May 2018) GBIF Occurrence Download <https://urldefense.proofpoint.com/v2/url?u=https-3A__doi.org_10.15468_dl.gcwutg&d=DwICAg&c=pZJPUDQ3SB9JplYbifm4nt2lEVG5pWx2KikqINpWlZM&r=9iE1-ZuRcrK1bcpQgDReSQ&m=Suu1AawbATtRZM4r2iPQSQ4CnYXwmRZ_8nJJvt1bNwc&s=0sMW7zXJOmGC4En_-bQlzbW7ZzAmeyQlTb77kLYfwEc&e=>

*Massonia depressa*

GBIF.org (31st May 2018) GBIF Occurrence Download <https://urldefense.proofpoint.com/v2/url?u=https-3A__doi.org_10.15468_dl.shoet5&d=DwICAg&c=pZJPUDQ3SB9JplYbifm4nt2lEVG5pWx2KikqINpWlZM&r=9iE1-ZuRcrK1bcpQgDReSQ&m=uDMbzV5GR4nN8jR5PpuPEIPkya_x8EO5nICsLvV-lqk&s=js9lhR6lJKnZspCnt7T58Yum7Wze3-LAK1hxsepfsSw&e=>

*Massonia echinata*

GBIF.org (29th May 2018) GBIF Occurrence Download <https://urldefense.proofpoint.com/v2/url?u=https-3A__doi.org_10.15468_dl.uvqvgh&d=DwICAg&c=pZJPUDQ3SB9JplYbifm4nt2lEVG5pWx2KikqINpWlZM&r=9iE1-ZuRcrK1bcpQgDReSQ&m=oIUp9SF4S49w8gLKycgyEP0_weUT8XXmkFcz_4p2wuU&s=JoJle8bzCVaPAb8m80vQ9uw9sYapzcsnWFCo7vvcLeA&e=>

*Muscari armeniacum*

GBIF.org (31st May 2018) GBIF Occurrence Download <https://urldefense.proofpoint.com/v2/url?u=https-3A__doi.org_10.15468_dl.yrdcli&d=DwICAg&c=pZJPUDQ3SB9JplYbifm4nt2lEVG5pWx2KikqINpWlZM&r=9iE1-ZuRcrK1bcpQgDReSQ&m=YBt4LShryJo6eUusPLLdI0m7A8m5Ybkrw9UkXI19uSo&s=i6zGHeIuJhmljyEPy915DrkqsZokNf3fgQpfBt6CsT8&e=>

*Muscari bourgaei*

GBIF.org (31st May 2018) GBIF Occurrence Download <https://urldefense.proofpoint.com/v2/url?u=https-3A__doi.org_10.15468_dl.vvfbmg&d=DwICAg&c=pZJPUDQ3SB9JplYbifm4nt2lEVG5pWx2KikqINpWlZM&r=9iE1-ZuRcrK1bcpQgDReSQ&m=KvDErvsQSYkjY6aCttrjgBOlesU4kAPX0NvBalTaaZs&s=z_A9LVxVCXj1sObW4uuFmrCGShOS6W6y645u1pB8_oc&e=>

*Muscari commutatum*

GBIF.org (31st May 2018) GBIF Occurrence Download <https://urldefense.proofpoint.com/v2/url?u=https-3A__doi.org_10.15468_dl.ejhbfd&d=DwICAg&c=pZJPUDQ3SB9JplYbifm4nt2lEVG5pWx2KikqINpWlZM&r=9iE1-ZuRcrK1bcpQgDReSQ&m=8ASsRGPEt3mDHK6XABQOhDOEejTczQkXsMb5wjoSR4w&s=Qm5gWNrb07Wf7s0qvv5-WceABu2WfG7mBT4-yBAy0SQ&e=>

*Narcissus bicolor*

GBIF.org (31st May 2018) GBIF Occurrence Download <https://urldefense.proofpoint.com/v2/url?u=https-3A__doi.org_10.15468_dl.9texkr&d=DwICAg&c=pZJPUDQ3SB9JplYbifm4nt2lEVG5pWx2KikqINpWlZM&r=9iE1-ZuRcrK1bcpQgDReSQ&m=IEILo6BMd-pLqOH05XwshCwDPRSHVsKWZSTq0IBcrXA&s=GnrpAB8noQ24hfKjshB4wU2JsOfTCH8qElQnGjz1G4w&e=>

*Narcissus dubius*

GBIF.org (31st May 2018) GBIF Occurrence Download <https://urldefense.proofpoint.com/v2/url?u=https-3A__doi.org_10.15468_dl.pklkur&d=DwICAg&c=pZJPUDQ3SB9JplYbifm4nt2lEVG5pWx2KikqINpWlZM&r=9iE1-ZuRcrK1bcpQgDReSQ&m=_r6DDD-vDYyu0JeFGr41Q23OegpgZFK7tAlOC_Vep7Y&s=2kRohadAHaDSJlq1XgTskEcbC_T88UWjJClkpN4yMZ0&e=>

*Narcissus minor*

GBIF.org (31st May 2018) GBIF Occurrence Download <https://urldefense.proofpoint.com/v2/url?u=https-3A__doi.org_10.15468_dl.eoipod&d=DwICAg&c=pZJPUDQ3SB9JplYbifm4nt2lEVG5pWx2KikqINpWlZM&r=9iE1-ZuRcrK1bcpQgDReSQ&m=wfXaKufbVf7RqDZvFek8P2SNd-jgEwO3GECcfHrQnPw&s=20fr_rHatM_G3YMaanoC8jeto81Z-Zgw19Q2kOVV258&e=>

*Narcissus pachybolbus*

GBIF.org (31st May 2018) GBIF Occurrence Download <https://urldefense.proofpoint.com/v2/url?u=https-3A__doi.org_10.15468_dl.ixq0fv&d=DwICAg&c=pZJPUDQ3SB9JplYbifm4nt2lEVG5pWx2KikqINpWlZM&r=9iE1-ZuRcrK1bcpQgDReSQ&m=-RaEs0IaTEWWeO9Tca5z25KeoJrhbucfNt7-0-wVmzg&s=el1nwh5bgU_e-WRLyQ5F_yk4W75zWBe3SwH7jbuHt1E&e=>

*Nothoscordum bivalve*

GBIF.org (31st August 2018) GBIF Occurrence Download <https://urldefense.proofpoint.com/v2/url?u=https-3A__doi.org_10.15468_dl.qdtu5y&d=DwICAg&c=pZJPUDQ3SB9JplYbifm4nt2lEVG5pWx2KikqINpWlZM&r=9iE1-ZuRcrK1bcpQgDReSQ&m=6AQgzZ05yu21epusKtxAJdB0FOB-l802wh7lgSPk29A&s=fBxO6Gut0d02smiyJ9iCZpRZTDFEpfp6T-_kS5yEyco&e=>

*Ornithogalum umbellatum*

GBIF.org (31st August 2018) GBIF Occurrence Download <https://urldefense.proofpoint.com/v2/url?u=https-3A__doi.org_10.15468_dl.s9xwhm&d=DwICAg&c=pZJPUDQ3SB9JplYbifm4nt2lEVG5pWx2KikqINpWlZM&r=9iE1-ZuRcrK1bcpQgDReSQ&m=uduB-F8PtIsQOocX3upqJec04f3KX4B1K8y2hLMil50&s=q4xHFx_wkFIqQRUBPY8kTGSSbzFr030aHj4x1aDvCt4&e=>

*Oziroe biflora*

GBIF.org (31st August 2018) GBIF Occurrence Download <https://urldefense.proofpoint.com/v2/url?u=https-3A__doi.org_10.15468_dl.phcjov&d=DwICAg&c=pZJPUDQ3SB9JplYbifm4nt2lEVG5pWx2KikqINpWlZM&r=9iE1-ZuRcrK1bcpQgDReSQ&m=3IXnBk6I77boEzr7pSGSd6zumfc0V2EkXvcGvQil7Y0&s=gCuOHxeFDJNajz-FvSwn1W-Ts9YCpEc5pNu-OmoH3gs&e=>

*Pancratium tenuifolium*

GBIF.org (31st May 2018) GBIF Occurrence Download <https://urldefense.proofpoint.com/v2/url?u=https-3A__doi.org_10.15468_dl.hrkqxc&d=DwICAg&c=pZJPUDQ3SB9JplYbifm4nt2lEVG5pWx2KikqINpWlZM&r=9iE1-ZuRcrK1bcpQgDReSQ&m=_j1ymv7gVivTx7xHTreffB7-J1ZCxL_ASLHoENZiV8k&s=pPrA4IB8DPAFkugiWnccwDSCmjH1lkk9CZbwKGJqhzc&e=>

*Phaedranassa dubia*

GBIF.org (31st August 2018) GBIF Occurrence Download <https://urldefense.proofpoint.com/v2/url?u=https-3A__doi.org_10.15468_dl.he3lln&d=DwICAg&c=pZJPUDQ3SB9JplYbifm4nt2lEVG5pWx2KikqINpWlZM&r=9iE1-ZuRcrK1bcpQgDReSQ&m=PksGLHo0YtayQSGYlgwYIjwrNoqbQXrbRFSomgNQPjg&s=u6J1cvqk8oA5I6Rfw9PY4yCtIGpcwE6MWNCMDI0XceI&e=>

*Placea arzae*

GBIF.org (31st May 2018) GBIF Occurrence Download <https://urldefense.proofpoint.com/v2/url?u=https-3A__doi.org_10.15468_dl.mxgz8z&d=DwICAg&c=pZJPUDQ3SB9JplYbifm4nt2lEVG5pWx2KikqINpWlZM&r=9iE1-ZuRcrK1bcpQgDReSQ&m=Nzr09X1pGkfYxuMrbUPJRcfeB82hEjDyjGi2-ivbohU&s=1kE-7SwuRo0MowWTxJT5QpyxUHmWHFcSbOtxmo_KoHg&e=>

*Poa bulbosa*

GBIF.org (29th May 2018) GBIF Occurrence Download <https://urldefense.proofpoint.com/v2/url?u=https-3A__doi.org_10.15468_dl.9dq3rw&d=DwICAg&c=pZJPUDQ3SB9JplYbifm4nt2lEVG5pWx2KikqINpWlZM&r=9iE1-ZuRcrK1bcpQgDReSQ&m=PhV0JaMoUGVy0-hGNiIPB_m_cB7ahbrAevspOew8iP8&s=Anafz0M13kRJKISwkSEFRE8kxgkOdIqxCnzMuI9SYOs&e=>

*Poa sinaica*

GBIF.org (31st May 2018) GBIF Occurrence Download <https://urldefense.proofpoint.com/v2/url?u=https-3A__doi.org_10.15468_dl.oxr1uk&d=DwICAg&c=pZJPUDQ3SB9JplYbifm4nt2lEVG5pWx2KikqINpWlZM&r=9iE1-ZuRcrK1bcpQgDReSQ&m=bmZUZoMIVQdlvCzsjpPY5BHtpsuQgeCUfsoK3ldMNUw&s=eGG0vMCCgqSlfqol1I7IZGF1KUa-bDSghd2QcwcwCkE&e=>

*Prospero autumnale*

GBIF.org (31st August 2018) GBIF Occurrence Download <https://urldefense.proofpoint.com/v2/url?u=https-3A__doi.org_10.15468_dl.re9ekx&d=DwICAg&c=pZJPUDQ3SB9JplYbifm4nt2lEVG5pWx2KikqINpWlZM&r=9iE1-ZuRcrK1bcpQgDReSQ&m=QOPiXKQc8jL_rfkCqvwSf2WLZXiFh4wGN3LVLlY21V4&s=cyTYsRb291ME323Zev0KOZyIrE37NPb5pxNuuJZFz8k&e=>

*Resnova humifusa*

GBIF.org (29th May 2018) GBIF Occurrence Download <https://urldefense.proofpoint.com/v2/url?u=https-3A__doi.org_10.15468_dl.nujypy&d=DwIFaQ&c=pZJPUDQ3SB9JplYbifm4nt2lEVG5pWx2KikqINpWlZM&r=9iE1-ZuRcrK1bcpQgDReSQ&m=wmVntyPzVSAU11o3trAKLafJNi1Kdbv75Hynv2WhFSo&s=0YyLcybNUBDYKqJUKSHQRYPjY9qRdC24adbWXUJYHvg&e=>

*Rhodophiala advena*

GBIF.org (31st May 2018) GBIF Occurrence Download <https://urldefense.proofpoint.com/v2/url?u=https-3A__doi.org_10.15468_dl.yg60qm&d=DwICAg&c=pZJPUDQ3SB9JplYbifm4nt2lEVG5pWx2KikqINpWlZM&r=9iE1-ZuRcrK1bcpQgDReSQ&m=8Sn7xxzn4zFr4CVIhfxzcnFf_MtLKFTx3lzfw_62UtQ&s=K76EA-PmsyMuJ-lgOeQ25vpKnJ_JxjoB926mucX4wmI&e=>

*Scadoxus multiflorus*

GBIF.org (28th August 2018) GBIF Occurrence Download <https://urldefense.proofpoint.com/v2/url?u=https-3A__doi.org_10.15468_dl.4ggmug&d=DwICAg&c=pZJPUDQ3SB9JplYbifm4nt2lEVG5pWx2KikqINpWlZM&r=9iE1-ZuRcrK1bcpQgDReSQ&m=IWNsZ4RiLi18_bNaJc0VraKdSCarAlsrb3lVdIT7Asc&s=h4UVWI2_gsT3M6WgIywznFszd8amBMBmW256fW9ScCA&e=>

*Schoenocaulon dubium*

GBIF.org (4th June 2018) GBIF Occurrence Download <https://urldefense.proofpoint.com/v2/url?u=https-3A__doi.org_10.15468_dl.uwcg7w&d=DwICAg&c=pZJPUDQ3SB9JplYbifm4nt2lEVG5pWx2KikqINpWlZM&r=9iE1-ZuRcrK1bcpQgDReSQ&m=kUKhuS0jL9a8IqtDFfFdvoZqLQQg6--AScgJD9nd1A8&s=QvONyZYyf1f6c2UxFF9NddzpI11fgwUkQdUmIAAsDZk&e=>

*Schoenocaulon ghiesbreghtii*

GBIF.org (4th June 2018) GBIF Occurrence Download [https://urldefense.proofpoint.com/v2/url?u=https-3A__doi.org_10.15468_dl.skc76c&d=DwICAg&c=pZJPUDQ3SB9JplYbifm4nt2lEVG5pWx2KikqINpWlZM&r=9iE1-ZuRcrK1bcpQgDReSQ&m=BSmtWqjXOWGO8uvrcb5Q5eQcOKOlUWP2GnpbkVDgm0I&s=HT4neXp2yQfvUq4skbrdMbORu_ZhY7w3x4Tj75vuw60&e](https://urldefense.proofpoint.com/v2/url?u=https-3A__doi.org_10.15468_dl.skc76c&d=DwICAg&c=pZJPUDQ3SB9JplYbifm4nt2lEVG5pWx2KikqINpWlZM&r=9iE1-ZuRcrK1bcpQgDReSQ&m=BSmtWqjXOWGO8uvrcb5Q5eQcOKOlUWP2GnpbkVDgm0I&s=HT4neXp2yQfvUq4skbrdMbORu_ZhY7w3x4Tj75vuw60&e=)

*Scilla hyacinthoides*

GBIF.org (31st May 2018) GBIF Occurrence Download <https://urldefense.proofpoint.com/v2/url?u=https-3A__doi.org_10.15468_dl.ffiror&d=DwICAg&c=pZJPUDQ3SB9JplYbifm4nt2lEVG5pWx2KikqINpWlZM&r=9iE1-ZuRcrK1bcpQgDReSQ&m=sXqisc1Y5qekMML0jWQK8q4Adbf2eYOyAViuFf8xViA&s=ypN2tKUwvZ7R4zYLo4MYZtkUWZXyrHLXTD9kVycTVI4&e=>

*Scilla monophyllos*

GBIF.org (31st May 2018) GBIF Occurrence Download <https://urldefense.proofpoint.com/v2/url?u=https-3A__doi.org_10.15468_dl.igriqf&d=DwICAg&c=pZJPUDQ3SB9JplYbifm4nt2lEVG5pWx2KikqINpWlZM&r=9iE1-ZuRcrK1bcpQgDReSQ&m=NgoNm552OYDfUwe1OSbuMZA7oxopY2KtGwk9WtX2MQE&s=JavSe3v0mJ1QBuBDkcnlkgnEQWSljdcpBXPtiAUtAMY&e=>

*Scilla nivalis*

GBIF.org (31st May 2018) GBIF Occurrence Download <https://urldefense.proofpoint.com/v2/url?u=https-3A__doi.org_10.15468_dl.usfo3u&d=DwICAg&c=pZJPUDQ3SB9JplYbifm4nt2lEVG5pWx2KikqINpWlZM&r=9iE1-ZuRcrK1bcpQgDReSQ&m=0pCzrBdaoAEvoJTM4vEGY2h5_Za_ePEtQsYNqDcIzyE&s=aoYZpAzuTMfiamHTtcVZ1z0VjMohEJcsQ4vxjFhVNCY&e=>

*Sprekelia formosissima*

GBIF.org (31st May 2018) GBIF Occurrence Download <https://urldefense.proofpoint.com/v2/url?u=https-3A__doi.org_10.15468_dl.mpovx3&d=DwICAg&c=pZJPUDQ3SB9JplYbifm4nt2lEVG5pWx2KikqINpWlZM&r=9iE1-ZuRcrK1bcpQgDReSQ&m=R6t0vrbsVqvXY2v8amqgoxKw_jYu0EqObrx2m_A5P08&s=5_lNt14kPaw-gt3VQ_B1La4FvenIeiCQb_xdqQGPUQI&e=>

*Toxicoscordion fremontii*

GBIF.org (4th June 2018) GBIF Occurrence Download <https://urldefense.proofpoint.com/v2/url?u=https-3A__doi.org_10.15468_dl.nakzua&d=DwICAg&c=pZJPUDQ3SB9JplYbifm4nt2lEVG5pWx2KikqINpWlZM&r=9iE1-ZuRcrK1bcpQgDReSQ&m=3AQPtgPhBMHGJNtQncLr1oZ7VbiLAY896icak8cFHow&s=uYCP51KIVqENo3l1JXAhXryMI3OFZbT4HysuAWQ2kCA&e=>

*Toxicoscordion paniculatum*

GBIF.org (4th June 2018) GBIF Occurrence Download <https://urldefense.proofpoint.com/v2/url?u=https-3A__doi.org_10.15468_dl.q7bfg2&d=DwICAg&c=pZJPUDQ3SB9JplYbifm4nt2lEVG5pWx2KikqINpWlZM&r=9iE1-ZuRcrK1bcpQgDReSQ&m=a5HqaNXiZ_yVnVdz7CWVH-vrghFV-lfbDp1-iGrX2mA&s=pNVORyjQf160H9Ez1ha46s9SFfaH7osVE85_Ym603oQ&e=>

*Tristagma nivale*

GBIF.org (31st August 2018) GBIF Occurrence Download <https://urldefense.proofpoint.com/v2/url?u=https-3A__doi.org_10.15468_dl.08ee4k&d=DwICAg&c=pZJPUDQ3SB9JplYbifm4nt2lEVG5pWx2KikqINpWlZM&r=9iE1-ZuRcrK1bcpQgDReSQ&m=8c84LXlT_RP6sOyiD7lYLjpkJzpnlSoE4bDpPIuG0yQ&s=KPMlyNnmkQ0praz69aZ2C9uqNyv2MT7KsnPjiQPNO9s&e=>

*Tulbaghia alliacea*

GBIF.org (31st May 2018) GBIF Occurrence Download <https://urldefense.proofpoint.com/v2/url?u=https-3A__doi.org_10.15468_dl.4ri93q&d=DwICAg&c=pZJPUDQ3SB9JplYbifm4nt2lEVG5pWx2KikqINpWlZM&r=9iE1-ZuRcrK1bcpQgDReSQ&m=JMRwF-bMaO5bxVcprR8thV-Chyicg6KiKlN7hvRCjew&s=5e-_C7RfnGy0M7-wfvqpYvzw20T_UE7t3sjrdap0bMk&e=>

*Tulipa agenensis*

GBIF.org (29th May 2018) GBIF Occurrence Download <https://urldefense.proofpoint.com/v2/url?u=https-3A__doi.org_10.15468_dl.ji0oa0&d=DwIFaQ&c=pZJPUDQ3SB9JplYbifm4nt2lEVG5pWx2KikqINpWlZM&r=9iE1-ZuRcrK1bcpQgDReSQ&m=SN7gkSzD3hWeaQCzRDxwjbTZYuvKYGe78hPigXXK97c&s=cqlKZq-KtraAQzJk2vSxQXt9cza7ctyXdvb8PxJv5VY&e=>

*Tulipa celsiana*

GBIF.org (31st May 2018) GBIF Occurrence Download <https://urldefense.proofpoint.com/v2/url?u=https-3A__doi.org_10.15468_dl.3htzhn&d=DwICAg&c=pZJPUDQ3SB9JplYbifm4nt2lEVG5pWx2KikqINpWlZM&r=9iE1-ZuRcrK1bcpQgDReSQ&m=3wDO83Fn_wRmmA-dupf_0hwUww7uvwOLyBIx2KxYhy0&s=AxjZb28s1-71z4RvFePu9JlAGm9riauBBuUrXtZ-OAg&e=>

*Tulipa gesneriana*

GBIF.org (31st May 2018) GBIF Occurrence Download <https://urldefense.proofpoint.com/v2/url?u=https-3A__doi.org_10.15468_dl.evq0br&d=DwICAg&c=pZJPUDQ3SB9JplYbifm4nt2lEVG5pWx2KikqINpWlZM&r=9iE1-ZuRcrK1bcpQgDReSQ&m=-FtiYksvT0qoJgWOYniJzhgZSvVzC_OXd_EZZGCerSc&s=wjG9DH8d0je0Iy22HppwqK9guxR5I71HTrrkWKdDM3I&e=>

*Zephyranthes candida*

GBIF.org (31st May 2018) GBIF Occurrence Download <https://urldefense.proofpoint.com/v2/url?u=https-3A__doi.org_10.15468_dl.rlh6gb&d=DwICAg&c=pZJPUDQ3SB9JplYbifm4nt2lEVG5pWx2KikqINpWlZM&r=9iE1-ZuRcrK1bcpQgDReSQ&m=lB2Pbfrf8XqKMRhnwFaf54inFiwHKwAbCAwsNExqOUU&s=x0fkNqvMf4Wsh_6Hb9c4l0FIkEzKmFNx05reXpNzNrw&e=>

*Zephyranthes treatiae*

GBIF.org (31st May 2018) GBIF Occurrence Download <https://urldefense.proofpoint.com/v2/url?u=https-3A__doi.org_10.15468_dl.hc012i&d=DwICAg&c=pZJPUDQ3SB9JplYbifm4nt2lEVG5pWx2KikqINpWlZM&r=9iE1-ZuRcrK1bcpQgDReSQ&m=MNOO54vUYpaG4d-jUC32_IvPnMXNAgQAWG-XOCUTtB4&s=6CkHmUYR7Dw2Ir3XyzfYoDjo2Qq0Cx4y-882SPlhiQI&e=>

*Allium cepa*

GBIF.org (31st January 2019) GBIF Occurrence Download <https://urldefense.proofpoint.com/v2/url?u=https-3A__doi.org_10.15468_dl.p3v82u&d=DwICAg&c=pZJPUDQ3SB9JplYbifm4nt2lEVG5pWx2KikqINpWlZM&r=9iE1-ZuRcrK1bcpQgDReSQ&m=8mkERf1PG5-cwQeP80wl1A5Hm--aVv7CJoQvSTxrY48&s=qG__wyiuGpfla5dDCKyVPD-_OU0UrY00htBb0W53Zs0&e=>

*Pancratium maritimum*

GBIF.org (31st January 2019) GBIF Occurrence Download <https://urldefense.proofpoint.com/v2/url?u=https-3A__doi.org_10.15468_dl.upwv3d&d=DwICAg&c=pZJPUDQ3SB9JplYbifm4nt2lEVG5pWx2KikqINpWlZM&r=9iE1-ZuRcrK1bcpQgDReSQ&m=o4OJ1kFM-Gm9dd0f0awOtgAmnKsRKcuca1Fhk20kx0U&s=h8nUdzkDla6JFzom_x7453CfyBovLsZ84PnI2bLwzsI&e=>

*Sternbergia lutea*

GBIF.org (31st January 2019) GBIF Occurrence Download <https://urldefense.proofpoint.com/v2/url?u=https-3A__doi.org_10.15468_dl.vi2crm&d=DwICAg&c=pZJPUDQ3SB9JplYbifm4nt2lEVG5pWx2KikqINpWlZM&r=9iE1-ZuRcrK1bcpQgDReSQ&m=FxaJcbHA25OyRD37jEee-mD5Lu7Zt_v4Z9BbXn9dK64&s=UgeBXfENhterk3qzYBJZohOuo1_RJKbX_RFBAx7r0gA&e=>

*Crinum jagus*

GBIF.org (31st January 2019) GBIF Occurrence Download <https://urldefense.proofpoint.com/v2/url?u=https-3A__doi.org_10.15468_dl.qlf3oh&d=DwICAg&c=pZJPUDQ3SB9JplYbifm4nt2lEVG5pWx2KikqINpWlZM&r=9iE1-ZuRcrK1bcpQgDReSQ&m=gkW_QZsBZoHseDDpTo64WYnnr9P904nQJ8aQSsgY5nQ&s=Ij7rYrEgedT6RQXAoM9jha7HaGFmwSO0xC6yuxQibps&e=>

*Pancratium trianthum*

GBIF.org (31st January 2019) GBIF Occurrence Download <https://urldefense.proofpoint.com/v2/url?u=https-3A__doi.org_10.15468_dl.acq15k&d=DwICAg&c=pZJPUDQ3SB9JplYbifm4nt2lEVG5pWx2KikqINpWlZM&r=9iE1-ZuRcrK1bcpQgDReSQ&m=poJZnMce7d4iu6jtdoEGGhgicmfslinGvukYw7dXnqo&s=Jvrunty2WTpcGmZOj-Wp-F526mS3eZNMaoSOu2HItxI&e=>

*Hymenocallis littoralis*

GBIF.org (31st January 2019) GBIF Occurrence Download <https://urldefense.proofpoint.com/v2/url?u=https-3A__doi.org_10.15468_dl.7wutmw&d=DwICAg&c=pZJPUDQ3SB9JplYbifm4nt2lEVG5pWx2KikqINpWlZM&r=9iE1-ZuRcrK1bcpQgDReSQ&m=dUHqYx32h3iwGVBcSlOS-Pu1Kqi5lunOG9-5N6zkD9M&s=SwkVZca52UngAcqWzDC_LhXWD_2HJ6TBcz6tulhr15I&e=>

*Crinum ornatum*

GBIF.org (31st January 2019) GBIF Occurrence Download <https://urldefense.proofpoint.com/v2/url?u=https-3A__doi.org_10.15468_dl.qedjuk&d=DwICAg&c=pZJPUDQ3SB9JplYbifm4nt2lEVG5pWx2KikqINpWlZM&r=9iE1-ZuRcrK1bcpQgDReSQ&m=LjUuBaqP2YJ6YVxaDO6qcYFi-DDy9sHJvKe6mbw81sU&s=K6X0gOrWs4oKUDxnJkz2dVDfX-fUZbdU46-bnAxoaEw&e=>

*sternbergia colchiciflora*

GBIF.org (31st January 2019) GBIF Occurrence Download <https://urldefense.proofpoint.com/v2/url?u=https-3A__doi.org_10.15468_dl.rpxtdj&d=DwICAg&c=pZJPUDQ3SB9JplYbifm4nt2lEVG5pWx2KikqINpWlZM&r=9iE1-ZuRcrK1bcpQgDReSQ&m=kZ8m2yV6ZMooxb9_Za-q3BkblIhJqrTf8xmSG9TBIsc&s=oPhkvLpkxIm3SVgMoaoT9hQsJokoifIyRhxrGqGPWpE&e=>

*Crinum americanum*

GBIF.org (31st January 2019) GBIF Occurrence Download <https://urldefense.proofpoint.com/v2/url?u=https-3A__doi.org_10.15468_dl.wurft4&d=DwICAg&c=pZJPUDQ3SB9JplYbifm4nt2lEVG5pWx2KikqINpWlZM&r=9iE1-ZuRcrK1bcpQgDReSQ&m=BvZCBtv0IlQAC8nO2Vt9Oe2W060qAscW6w9XVNkjaZ8&s=8PnI80MaFD52e46HS4-8FjUP2OWozoh-LQw7cE1pqrY&e=>

*Sternbergia clusiana*

GBIF.org (31st January 2019) GBIF Occurrence Download <https://urldefense.proofpoint.com/v2/url?u=https-3A__doi.org_10.15468_dl.piwypo&d=DwICAg&c=pZJPUDQ3SB9JplYbifm4nt2lEVG5pWx2KikqINpWlZM&r=9iE1-ZuRcrK1bcpQgDReSQ&m=_ecgGzSlNeavysA1xSIc4WiP9_CSZbwid1F9ivAu_eg&s=de9-oVjEnhTqqau06eByEgtqE_jTRy9H9T-odmYm9Kg&e=>

*Ammocharis longifolia*

GBIF.org (31st January 2019) GBIF Occurrence Download <https://urldefense.proofpoint.com/v2/url?u=https-3A__doi.org_10.15468_dl.s41pap&d=DwICAg&c=pZJPUDQ3SB9JplYbifm4nt2lEVG5pWx2KikqINpWlZM&r=9iE1-ZuRcrK1bcpQgDReSQ&m=dszWZaPakCdl5_-QLxczrNy-HOaOj72tMxA7nvjjgd0&s=6rKQVouXW_trrLGKLOpi0mIE8NgB8DTFtxH5UpZ4YkU&e=>

*Drimia maritima*

GBIF.org (31st January 2019) GBIF Occurrence Download <https://urldefense.proofpoint.com/v2/url?u=https-3A__doi.org_10.15468_dl.3hvc4r&d=DwICAg&c=pZJPUDQ3SB9JplYbifm4nt2lEVG5pWx2KikqINpWlZM&r=9iE1-ZuRcrK1bcpQgDReSQ&m=d68wJTWI9SmC8HpvGpw1S07o0z6Rhb0KyfYO8lBL4mY&s=jIE3F9v8f7MVOPzS_2CwU1yjFDmTxsDekBlbxaXcmxA&e=>

*Drimia altissima*

GBIF.org (31st January 2019) GBIF Occurrence Download <https://urldefense.proofpoint.com/v2/url?u=https-3A__doi.org_10.15468_dl.sggvhb&d=DwICAg&c=pZJPUDQ3SB9JplYbifm4nt2lEVG5pWx2KikqINpWlZM&r=9iE1-ZuRcrK1bcpQgDReSQ&m=xYQlXciBIzXfCxUzbJgFyXU1k9Y8XK9tePlugigcPjI&s=LliLz6Aj4puWsSDQWilIb7Srw3qfCf50cdQBZrtM4EA&e=>

*drimia undata*

GBIF.org (31st January 2019) GBIF Occurrence Download <https://urldefense.proofpoint.com/v2/url?u=https-3A__doi.org_10.15468_dl.usxbam&d=DwICAg&c=pZJPUDQ3SB9JplYbifm4nt2lEVG5pWx2KikqINpWlZM&r=9iE1-ZuRcrK1bcpQgDReSQ&m=F-mOnmJbLBV8Ii-36R_SKFgJgklL85uJZvBT0EX4URs&s=LEE_PtTsLKyPKJjCAzSolo4ncKGrhspofIbY96pzhoQ&e=>

*Drimia intricata*

GBIF.org (31st January 2019) GBIF Occurrence Download <https://urldefense.proofpoint.com/v2/url?u=https-3A__doi.org_10.15468_dl.bcrckz&d=DwICAg&c=pZJPUDQ3SB9JplYbifm4nt2lEVG5pWx2KikqINpWlZM&r=9iE1-ZuRcrK1bcpQgDReSQ&m=rHnXqlblNmiK1A0bLjdhKFEUo_A_Hr8DVi1Az5lZOQ0&s=gawbft6HyWfSAdMEuSqK5ogxRTnutCRCVVoyLuT1JDM&e=>

*Haemodorum brevisepalum*

GBIF.org (31st January 2019) GBIF Occurrence Download <https://urldefense.proofpoint.com/v2/url?u=https-3A__doi.org_10.15468_dl.yrzv1r&d=DwICAg&c=pZJPUDQ3SB9JplYbifm4nt2lEVG5pWx2KikqINpWlZM&r=9iE1-ZuRcrK1bcpQgDReSQ&m=LpFHm34q8xseyp01b6bqCBOnaOs70NZL-r1lxB5MM0o&s=ONKeOM6HUL4P7XwJlxUv-HIUae8LL5FBEP-mC1BRAq0&e=>

*Hyacinthus orientalis*

GBIF.org (31st January 2019) GBIF Occurrence Download <https://urldefense.proofpoint.com/v2/url?u=https-3A__doi.org_10.15468_dl.nus94g&d=DwICAg&c=pZJPUDQ3SB9JplYbifm4nt2lEVG5pWx2KikqINpWlZM&r=9iE1-ZuRcrK1bcpQgDReSQ&m=yG0FOTQkpV2L4Yx7OwA5HYHG6JHUQnwHT6K9Ufwl_hY&s=4mYNIGT7RTRVmyUmI1-Bt9OGGdRq_lRT4-p7kpj7pTc&e=>
