# Supplementary figures and images for: Tunicate bulb size variation in monocots explained by temperature and phenology
Source: Ecol Evol. 2020 Feb 27;10(5):2299–309. doi: 10.1002/ece3.5996 (PMC7069286; doi:10.1002/ece3.5996)

# Climatic variables grouped by bulb type

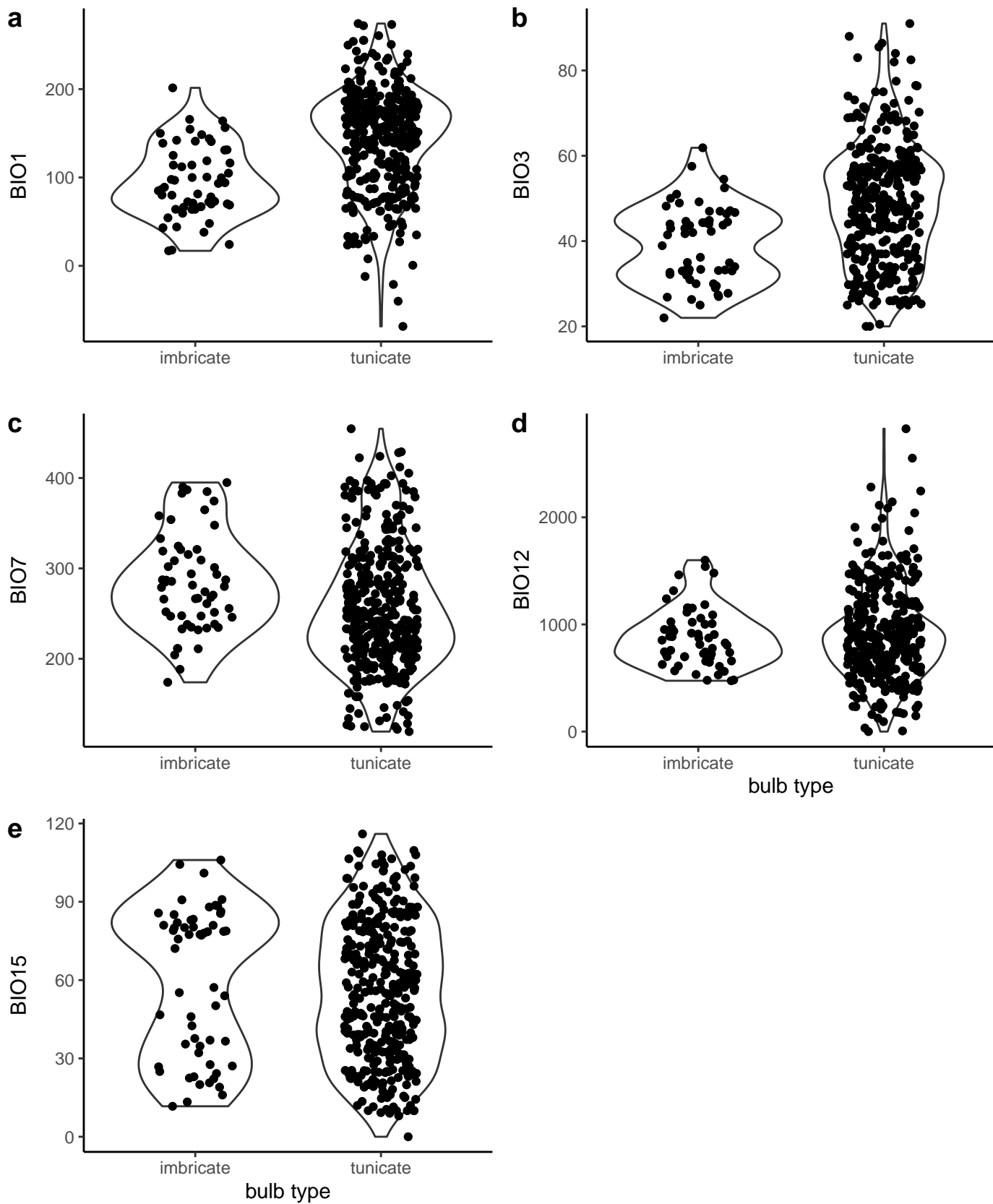

Supplement: Supplementary file 2 [file ECE3-10-2299-s002.pdf]

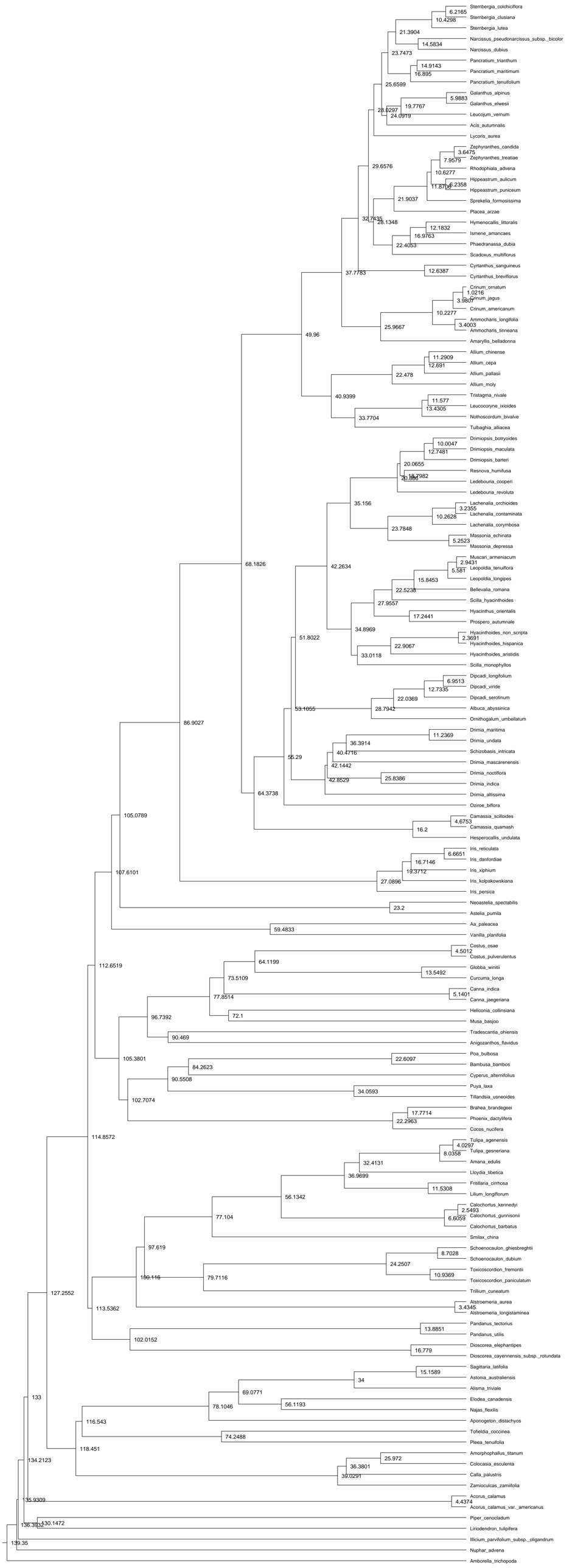

Supplement: Supplementary file 3 [file ECE3-10-2299-s003.pdf]

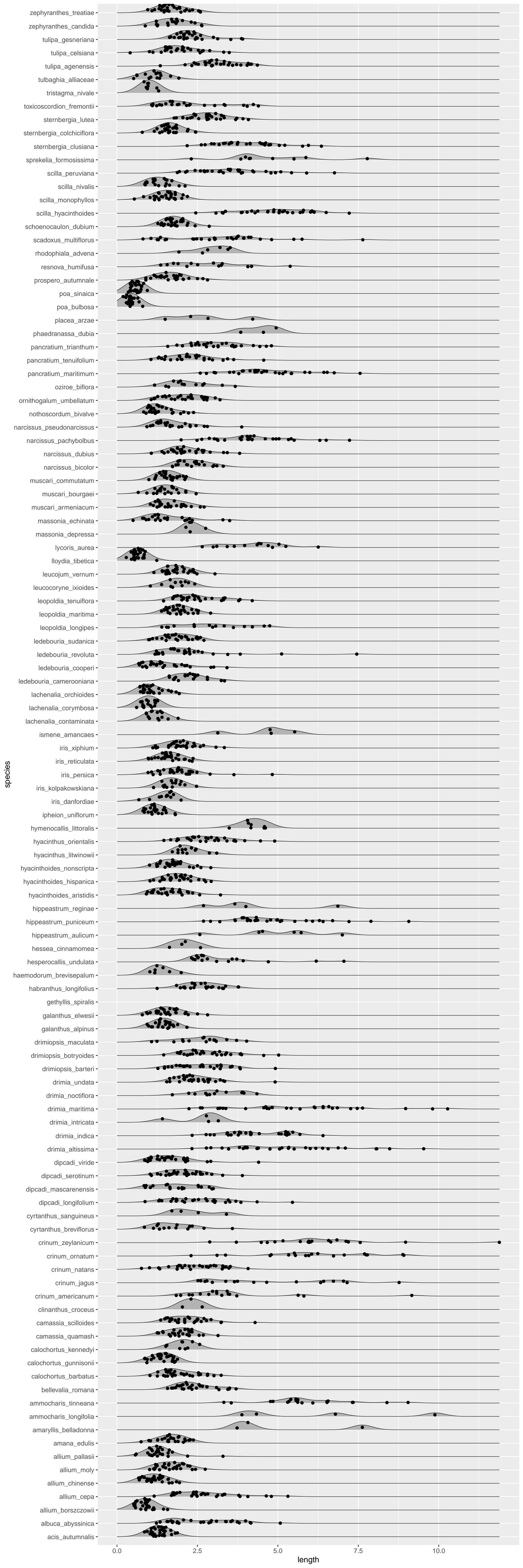

Supplement: Supplementary file 4 [file ECE3-10-2299-s004.pdf]

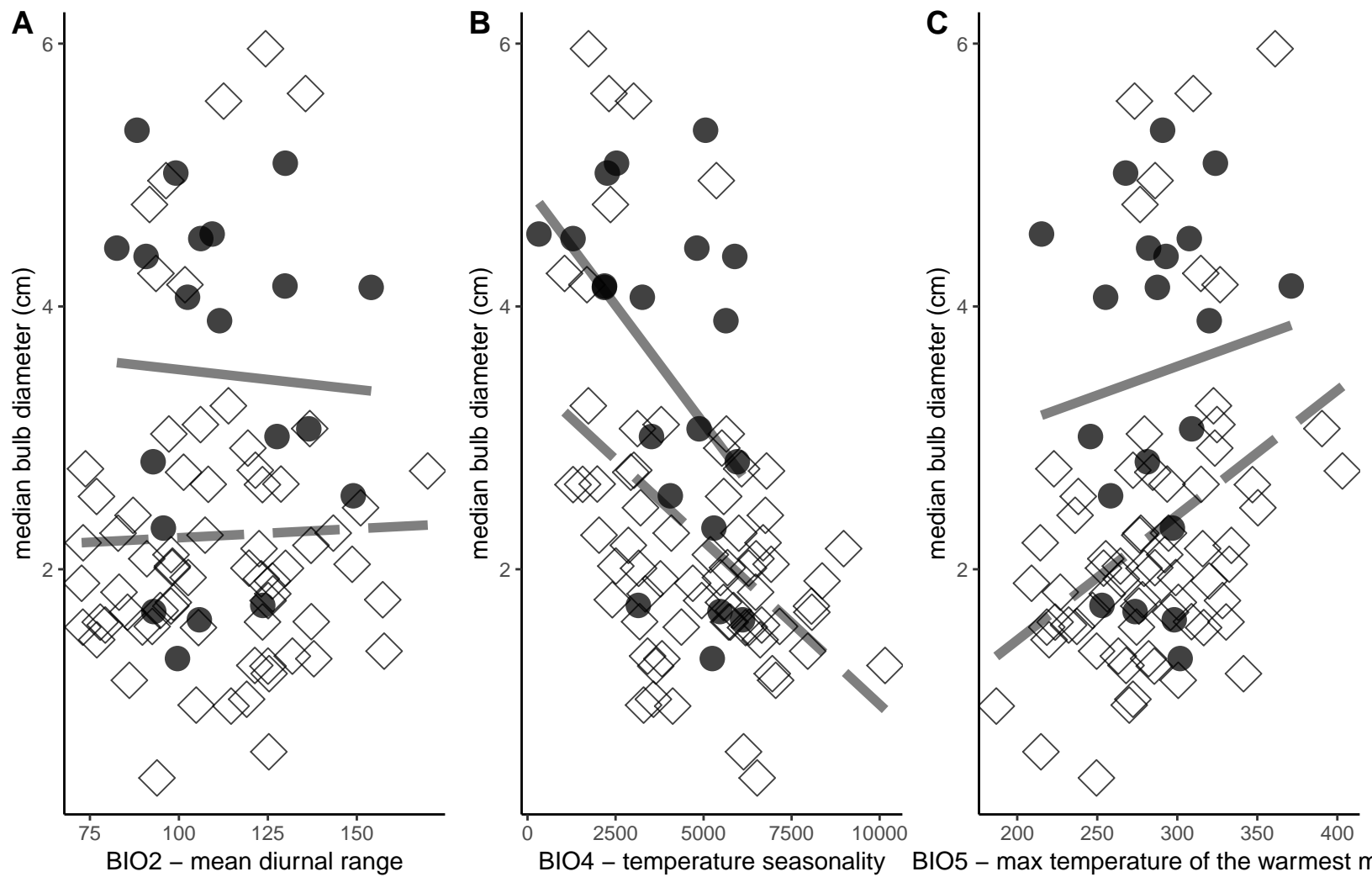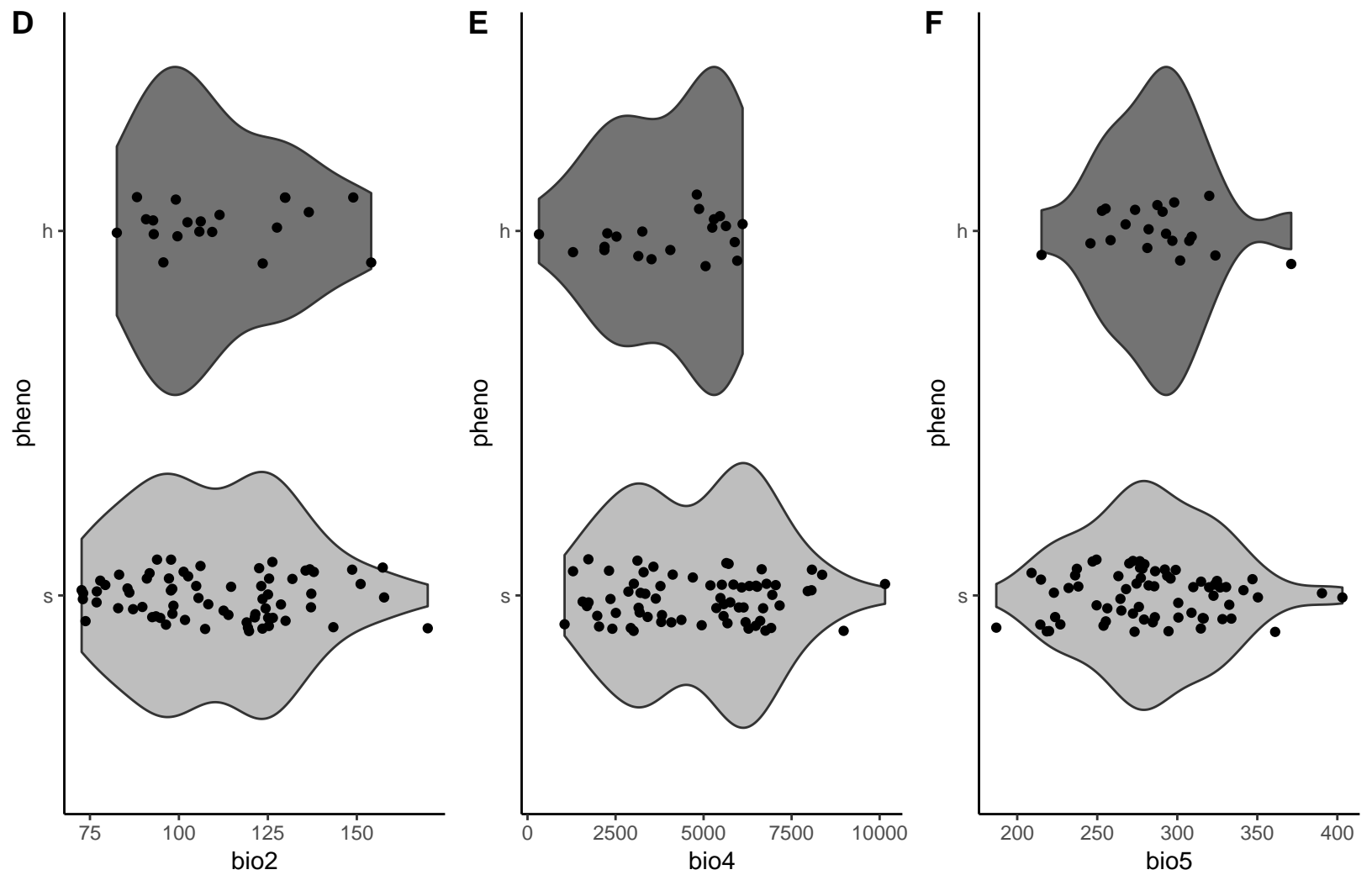

Supplement: Supplementary file 6 [file ECE3-10-2299-s006.pdf]

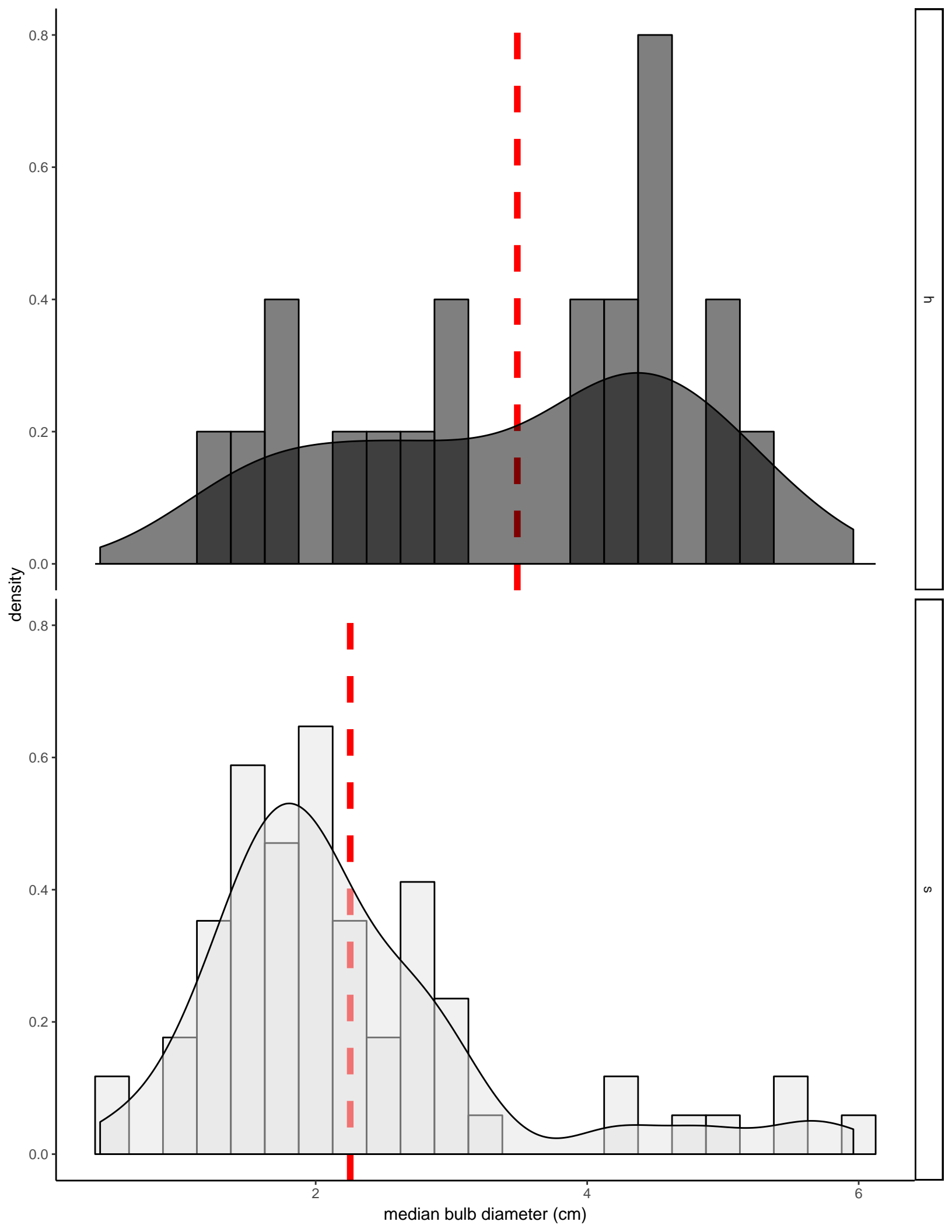

Supplement: Supplementary file 7 [file ECE3-10-2299-s007.pdf]
